# The genetics of folate metabolism and maternal risk of birth of a child with Down syndrome and associated congenital heart defects

**DOI:** 10.3389/fgene.2015.00223

**Published:** 2015-06-25

**Authors:** Fabio Coppedè

**Affiliations:** ^1^Section of Medical Genetics, Department of Translational Research and New Technologies in Medicine and Surgery, University of PisaPisa, Italy; ^2^Interdepartmental Research Center Nutrafood “Nutraceuticals and Food for Health,” University of PisaPisa, Italy

**Keywords:** folate, polymorphisms, Down syndrome, congenital heart defects, DNA methylation, epigenetics, MTHFR, folic acid supplementation

## Abstract

Almost 15 years ago it was hypothesized that polymorphisms of genes encoding enzymes involved in folate metabolism could lead to aberrant methylation of peri-centromeric regions of chromosome 21, favoring its abnormal segregation during maternal meiosis. Subsequently, more than 50 small case-control studies investigated whether or not maternal polymorphisms of folate pathway genes could be risk factors for the birth of a child with Down syndrome (DS), yielding conflicting and inconclusive results. However, recent meta-analyses of those studies suggest that at least three of those polymorphisms, namely *MTHFR* 677C>T, *MTRR* 66A>G, and *RFC1* 80G>A, are likely to act as maternal risk factors for the birth of a child with trisomy 21, revealing also complex gene-nutrient interactions. A large-cohort study also revealed that lack of maternal folic acid supplementation at peri-conception resulted in increased risk for a DS birth due to errors occurred at maternal meiosis II in the aging oocyte, and it was shown that the methylation status of chromosome 21 peri-centromeric regions could favor recombination errors during meiosis leading to its malsegregation. In this regard, two recent case-control studies revealed association of maternal polymorphisms or haplotypes of the *DNMT3B* gene, coding for an enzyme required for the regulation of DNA methylation at centromeric and peri-centromeric regions of human chromosomes, with risk of having a birth with DS. Furthermore, congenital heart defects (CHD) are found in almost a half of DS births, and increasing evidence points to a possible contribution of lack of folic acid supplementation at peri-conception, maternal polymorphisms of folate pathway genes, and resulting epigenetic modifications of several genes, at the basis of their occurrence. This review summarizes available case-control studies and literature meta-analyses in order to provide a critical and up to date overview of what we currently know in this field.

## Introduction

Two studies conducted nearly 15 years ago in North America (James et al., 1999; Hobbs et al., 2000) have suggested that polymorphisms of genes encoding enzymes involved in the folate metabolic pathway, also known as one-carbon metabolism, may be maternal risk factors for the birth of a child with Down syndrome (DS). In particular, it was hypothesized that an altered metabolism of folate, resulting from the presence of polymorphisms in metabolic genes, could lead to aberrant methylation of peri-centromeric regions of chromosome 21, favoring its abnormal segregation during maternal meiosis and leading to the formation of eggs with two copies of chromosome 21, which, if fertilized, would result in a zygote with full trisomy for chromosome 21 (James et al., 1999; Hobbs et al., 2000). Those works were followed by more than 50 small case-control studies all aimed to address this issue, listed in Table 1. Overall, most of those papers are in favor of a possible contribution of polymorphisms in folate-related genes as maternal risk factors for the birth of a child with DS, however, they are often conflicting and limited by the small sample size of case-control cohorts (Table 1). In 2009, I reviewed the literature in the field, concluding that at that time no single gene could be clearly linked to the maternal risk of birth of a child with DS, due to the relatively small sample-size and the conflicting nature of the findings among the different studies (Coppedè, 2009). I also recommended collaboration among research groups and/or literature meta-analyses in order to increase the power to detect association (Coppedè, 2009). Many papers have been produced between 2009 and present days (Table 1), and literature meta-analyses are nowadays available overall suggesting that at least three polymorphisms of folate pathway genes are likely to be associated to the maternal risk for trisomy 21 (Table 2), while an increasing number of studies also suggest complex gene-gene and gene-nutrient interactions (Tables 1, 3). However, the original hypothesis linking impaired folate metabolism and abnormal methylation levels of chromosome 21 peri-centromeric regions (James et al., 1999) has not yet been demonstrated, even if *in vitro* studies revealed that cells under folate deprivation increase the rate of chromosome 21 aneuploidy (Wang et al., 2004; Beetstra et al., 2005), and recent evidence suggests that DNA methylation levels of peri-centromeric regions of chromosome 21 might be responsible of recombination errors leading to its malsegregation during meiosis (Oliver et al., 2014). Moreover, that hypothesis (James et al., 1999) has been revised and implemented over the years, leading researchers to hypothesize that maternal polymorphisms of genes involved in folate metabolism could not only favor chromosome 21 malsegregation but, when transmitted to a DS embryo, could also account for the probability that it will survive up to the birth (Martínez-Frías et al., 2006), or contribute to the onset of congenital defects (Brandalize et al., 2009; Locke et al., 2010), such as congenital heart defects (CHD) (Table 4). This article summarizes both case-control studies and literature meta-analyses in order to provide a critical and up to date review of what we currently know about maternal polymorphisms of folate-related genes and risk of birth of a child with DS and associated CHD, highlighting the strengths and the limitations of our current knowledge in this field.

**Table 1:** **Genetic association studies of folate pathway genes as maternal risk factors for having a child with Down syndrome**.

**References**	**Country**	**MDS/MC^a^**	**Studied polymorphism/s**	**Significant findings**
James et al., 1999	USA Canada	57/50	*MTHFR* 677C>T	*MTHFR* 677CT (OR = 2.5; 95% CI = 1.0–5.7) *MTHFR* 677CT + TT (OR = 2.6; 95% CI = 1.2–5.8)
Hobbs et al., 2000	USA Canada	157/144	*MTHFR* 677C>T *MTRR* 66A>G	*MTHFR* 677CT (OR = 1.9; 95% CI = 1.1–3.1) *MTHFR* 677CT + TT (OR = 1.9; 95% CI = 1.2–3.0) *MTRR* 66GG (OR = 2.6; 95% CI = 1.3–5.0) *MTRR* 66AG + GG (OR = 1.8; 95% CI = 1.0–3.1) *MTHFR* 677TT or CT + *MTRR* 66GG (OR = 4.1; 95% CI = 1.9–8.6)
O'Leary et al., 2002	Ireland	48/192	*MTHFR* 677C>T *MTRR* 66A>G	*MTRR* 66AG (OR = 8.0; 95% CI = 1.0–61.2) *MTRR* 66GG (OR = 15; 95% CI = 1.9–116) *MTRR* 66AG + GG (OR = 10.5; 95% CI = 1.4–78.6) *MTHFR* 677CT or TT + *MTRR* 66GG (OR = 3.0; 95% CI = 1.2–7.5)
Chadefaux-Vekemans et al., 2002	France	85/107	*MTHFR* 677C>T	No association observed
Grillo et al., 2002	Brazil	36/200	*MTHFR* 677C>T *MTHFR* 1298A>C	*MTHFR* 677CT + *MTHFR* 1298AC associated with increased maternal risk OR = N.A.
Stuppia et al., 2002	Italy	64/112	*MTHFR* 677C>T	No association observed
Bosco et al., 2003	Italy	63/72	*MTHFR* 677C>T *MTHFR* 1298A>C *MTRR* 66A>G *MTR* 2756A>G	*MTR* 2756AG + GG (OR = 3.5; 95% CI = 1.2–10.9) *MTR* 2756AG + *MTRR* 66AG (OR = 5.0; 95% CI = 1.1–24.1)
Takamura et al., 2004	Japan	31/60	*MTHFR* 677C>T	No association observed
Boduroğlu et al., 2004	Turkey	152/91	*MTHFR* 677C>T *MTHFR* 1298A>C	No association observed
da Silva et al., 2005	Brazil	154/158	*MTHFR* 677C>T *MTHFR* 1298A>C *MTRR* 66A>G *MTR* 2756A>G *CBS* 844 ins68	*MTHFR* 677T allele (OR = 1.4; 95% CI = 1.0–2.1) The presence of increasing numbers of the variant alleles (*MTHFR* 677T, *MTHFR* 1298C, *MTR* 2756G, *MTRR* 66G, and *CBS* 844ins68) increases maternal risk (OR = 1.2; 95 CI = 1.0–1.6)
Chango et al., 2005	France	119/119	*MTHFR* 677C>T *MTHFR* 1298A>C *MTRR* 66A>G *MTR* 2756A>G *CBS* 844 ins68 *RFC1* 80G>A	No association observed
Acácio et al., 2005	Brazil	70/88	*MTHFR* 677C>T *MTHFR* 1298A>C	*MTHFR* 677CT + *MTHFR* 1298AC (OR = 5.7; 95% CI = 1.7–18.8)
Coppedè et al., 2006	Italy	80/111	*MTHFR* 677C>T *MTHFR* 1298A>C *RFC1* 80G>A	*MTHFR* 677TT + *RFC1* 80GG (OR = 6.0; 95% CI = 1.0–35.9) *MTHFR* 1298AA + *RFC1* 80GA/AA (OR = 0.4; 95% CI = 0.1–0.9)
Rai et al., 2006	India	149/165	*MTHFR* 677C>T *MTHFR* 1298A>C	*MTHFR* 677TT (OR = 7.7; 95% CI = 1.7–35.1) *MTHFR* 1298CC (OR = 4.4; 95% CI = 1.4–13.3) *MTHFR* 677CC + *MTHFR* 1298CC (OR = 4.1; 95% CI = 1.2–13.7)
Scala et al., 2006	Italy	94/264	*MTHFR* 677C>T *MTHFR* 1298A>C *MTRR* 66A>G	*MTHFR* 1298C allele (OR = 1.5; 95% CI = 1.0–2.1) *RFC1* 80G allele (OR = 1.5; 95% CI = 1.0–2.1) *MTHFR* 1298CC (OR = 2.3; 95% CI = 1.1–5.0)
			*MTR* 2756A>G *CBS* 844 ins68 *RFC1* 80G>A *MTHFD1* 1958G>A	*RFC1* 80GG (OR = 2.0; 95% CI =1.0−4.1) *MTHFR* 677TT + *MTHFR* 1298CC or CA (OR = 7.2; 95% CI = 1.4–47.2) *MTHFR* 1298CC or CA + *RFC1* 80GG or GA (OR = 2.6; 95% CI =1.1–6.3) *RFC1* 80GG + *MTHFD1* 1958AA (OR = 4.4; 95% CI = 1.2–17.9)
Wang et al., 2007	China	100/100	*MTHFR* 677C>T	*MTHFR* 677CT (OR = 2.1; 95% CI = 1.1–3.9) *MTHFR* 677TT (OR = 3.4; 95% CI = 1.4–8.4)
Meguid et al., 2008	Egypt	42/48	*MTHFR* 677C>T *MTHFR* 1298A>C	*MTHFR* 677CT +TT (OR = 4.1; 95% CI = 1.0–5.7) *MTHFR* 1298CC (OR = 31.5; 95% CI = 3.5–282.)
Martínez-Frías, 2008	Spain	146/188	*MTHFR* 677C>T *MTHFR* 1298A>C	No association observed
Wang et al., 2008	China	64/70	*MTHFR* 677C>T *MTRR* 66A>G	*MTHFR* 677TT (OR = 9.3; 95% CI = 2.9–29.7) *MTRR* 66GG (OR = 5.2; 95% CI = 1.9–14.2) *MTHFR* 677TT/CT + *MTRR* 66GG (OR = 6.0; 95% CI =2.0–17.5)
Biselli et al., 2008a	Brazil	67/113	*RFC1* 80G>A *TCN2* 776C>G	No association observed
Biselli et al., 2008b	Brazil	72/194	*MTHFR* 677C>T *MTHFR* 1298A>C *MTR* 2756A>G *RFC1* 80G>A	The presence of increasing numbers of 3 or more polymorphic alleles among *MTHFR* 677T, *MTHFR* 1298C, *MTR* 2756G, and *RFC1* 80G, increases maternal risk (OR = 1.7; 95% CI = 1.0–3.0)
Kohli et al., 2008	India	104/109	*MTHFR* 677C>T	No association observed
Santos-Rebouças et al., 2008	Brazil	103/108	*MTHFR* 677C>T *MTHFR* 1298A>C *MTRR* 66A>G	No association observed
Coppedè et al., 2009	Italy	94/113	*MTHFR* 677C>T *MTHFR* 1298A>C *MTRR* 66A>G *MTR* 2756A>G *TYMS* 28bp 2R/3R *TYMS* 1494 ins/del	*MTHFR* 677TT + *MTR* 2756AA (OR = 3.0; 95% CI = 1.0–8.5) *MTHFR* 1298AC + *TYMS* 2R/2R (OR = 0.11; 95% CI = 0.1–0.5)
Pozzi et al., 2009	Italy	74/184	*MTHFR* 677C>T *MTRR* 66A>G	*MTRR* 66AG (OR = 2.6; 95% CI = 1.2–5.5) *MTRR* 66AG + GG (OR = 2.2; 95% CI = 1.1–4.4)
Fintelman-Rodrigues et al., 2009	Brazil	114/110	*RFC1* 80G>A *TCN2* 776C>G *MTR* 2756A>G *CBS* 844 ins68	*MTR* 2756AG/*TCN2* 776CC (OR = 3.2; 95% CI = 1.1–9.0)
Cyril et al., 2009	India	36/60	*MTHFR* 677C>T *MTHFR* 1298A>C	*MTHFR* 677 (CT+TT) (OR = 12.6; 95% CI = 6.5–99.7)
Kokotas et al., 2009	Denmark	181/1084	*MTHFR* 677C>T	No association observed
Brandalize et al., 2009	Brazil	239/197	*MTHFR* 677C>T *MTHFR* 1298A>C	*MTHFR* 677CT or TT/*MTHFR* 1298AA (OR = 1.9; 95% CI = 1.1–3.5)
Brandalize et al., 2010	Brazil	239/197	*MTR* 2756A>G *MTRR* 66A>G *RFC1* 80G>A *CBS* 844 ins68	Combined genotypes among *MTR* 2756A>G, *MTRR* 66A>G, *CBS* 844ins68, and *RFC1* 80G>A polymorphisms increase maternal risk. ORs ranging from 4.8 to 6.9 depending on the number of risk alleles considered. * MTHFR* 677T(from Brandalize et al., 2009) + *MTRR* 66G (OR = 1.5; 95% CI = 1.0–2.3)
Liao et al., 2010	China	60/68	*MTHFR* 677C>T *MTRR* 66A>G *MTR* 2756A>G *RFC1* 80G>A	*MTHFR* 677TT (OR = 3.5; 95% CI = 1.3–9.5) *MTRR* 66GG (OR = 3.2; 95% CI = 1.2–8.3) *MTHFR* 677 (CT or TT) + *MTRR* 66GG (OR 9.5; 95% CI = 2.1–46.3) *MTHFR* (CT or TT) + *RFC-1* AA (OR 5.2; 95% CI = 1.7–15.9) *MTHFR* 677CC + *MTR* 2756(AG or GG) (OR = 6.7; 95% CI = 1.2–35.0) *MTHFR* 677 (CT or TT) + *MTR* 2756 AA (OR = 4.2; 95% CI = 1.5–11.6) *MTRR* 66GG + *MTR* 2756AA (OR = 3.0; 95% CI = 1.1–8.7) *RFC1* 80AA + *MTR* 2756AA (OR = 2.7; 95% CI = 1.2–6.5)
Neagos et al., 2010a	Romania	26/46	*RFC1* 80G>A	No association observed
Mendes et al., 2010	Brazil	105/184	*DHFR* 19 bp ins/del	No association observed
Neagos et al., 2010b	Romania	26/46	*MTHFD1* 1958G>A	No association observed
Coppedè et al., 2010	Italy	29/32	*MTHFR* 677C>T *MTHFR* 1298A>C *MTRR* 66A>G *MTR* 2756A>G *RFC1* 80G>A *TYMS* 28 bp 2R/3R *TYMS* 1494 ins/del	Artificial neural networks selected 6 variables (micronucleus frequency, *MTHFR* 677TT, *RFC1* 80AA, *TYMS* 1494 6bp +/+, *TYMS* 28 bp 3R/3R and *MTR* 2756AA genotypes) that allowed to discriminate between MDS and control mothers with 90% accuracy
Vraneković et al., 2010	Croatia	111/141	*MTHFR* 677C>T *MTHFR* 1298A>C	No association observed
Sadiq et al., 2011	Jordan	53/29	*MTHFR* 677C>T *MTHFR* 1298A>C	*MTHFR* 677T allele (OR 3.1; 95% CI = 1.3–7.7)
Marucci et al., 2012	Brazil	105/185	*SHMT* 1420C>T	*SHMT* 1420CC (OR = 0.35; 95% CI = 0.2–0.6) *SHMT* 1420CT (OR = 0.2; 95 % CI = 0.1–0.4)
Zampieri et al., 2012a	Brazil	86/161	*MTHFD1* 1958G>A *BHMT* 742A>G	*BHMT* 742AA (OR = 0.3; 95% CI = 0.1–0.9) *BHMT* 742 (GA + AA) (OR = 0.6; 95% CI = 0.4–0.9)
Zampieri et al., 2012b	Brazil	105/185	*MTHFR* 677C>T *MTHFR* 1298A>C *MTHFR* 1317T>C *MTR* 2756A>G *MTRR* 66A>G *RFC1* 80G>A *TCN2* 67A>G *TCN2* 776C>G *CBS* 844ins68 *CBS* 833T>C *MTHFD1* 1958G>A *BHMT* 742G>A	*MTHFR* 677CT or TT (OR = 1.8; 95% CI = 1.0–3.1) *TCN2* 776GG (OR = 2.4; 95% CI = 1.0–5.8) *BHMT* 742AA (OR = 0.3; 95% CI = 0.1–0.8) *MTHFR* 677C/1298A/1317T haplotype (more frequent in control mothers *P* = 0.01)
Mohanty et al., 2012	India	52/52	*MTHFR* 677C>T	No association observed
Tayeb, 2012	Saudi Arabia	30/40	*MTHFR* 677C>T	No association observed
Wang et al., 2013a	China	104/184	*MTRR* 524C>T	*MTRR* 524TT (OR = 0.3; 95% CI = 0.1–0.9) *MTRR* 524 (CT + TT) (OR = 0.6; 95% CI = 0.4–1.0)
Wang et al., 2013b	China	104/184	*RFC1* 80G>A *CBS* 833T>C	*RFC1* 80G allele (OR 1.5; 95% CI = 1.0–2.2) *CBS* 833C (OR 1.5; 95% CI = 1.1–2.2) *RFC1* 80GG + *CBS* 833TC (OR 4.8; 95% CI = 1.8–12.7)
Amorim et al., 2013	Brazil	94/134	*BHMT* 742G>A	*BHMT* 742A (OR = 0.6; 95% CI = 0.4–0.9) *BHMT* 742AA (OR = 0.2; 95% CI = 0.04–0.8)
Coppedè et al., 2013a	Italy	286/305	*MTR* 2756A>G	No association observed
Coppedè et al., 2013b	Italy	172/157	*DNMT3B* −579G>T *DNMT3B* −149C>T	*DNMT3B* −579T (OR 0.7; 95% CI = 0.5–0.9) *DNMT3B* −579GT (OR 0.5; 95% CI = 0.3–0.9) *DNMT3B* −579 (GT+TT) (OR 0.5; 95% CI = 0.3–0.9) *DNMT3B* −579GT + *DNMT3B* −149CC (OR = 0.2; 95% CI = 0.1–0.6)
Kaur and Kaur, 2013	India	110/111	*MTHFR* 677C>T	No association observed
Cretu et al., 2013	Romania	26/46	*CBS* 844ins68	No association observed
Pandey et al., 2013	India	80/100	*MTHFR* 677C>T *MTHFR* 1298A>C	*MTHFR* 1298 (AC+CC) (OR= 3.1, 95% CI 1.6–5.8)
Liao et al., 2014	China	76/115	*MTHFD1* 1958G>A *TCN2* 776C>G	*MTHFR* 677CT/TT + *MTHFD1* 1958AA/GA (OR = 3.1; 95% CI = 1.1–9.0) *TCN2* 776CG + *MTHFR* 677TT (OR = 3.6; 95% CI = 1.3–10.3) *MTHFR* data taken from Liao et al. (2010)
Coppedè et al., 2014	Italy	253/298	*MTRR* 66A>G	*MTR* 2756AG + *MTRR* 66AG (OR 1.8; 95 % CI = 1.0–3.2) *MTR* data taken from Coppedè et al. (2013a)
Jaiswal et al., 2015	India	150/172	*DNMT3B* -579G>T *DNMT3B* -149C>T	Significant increased frequency of the -579G/-149T haplotype in case mothers (*P* = 0.002)
Izci Ay et al., 2015	Turkey	47/49	*MTHFR* 677C>T *MTHFR* 1298A>C *MTRR* 66A>G *RFC1* 80G>A *CBS* 844ins68 *MTHFD1* 1958G>A	*MTHFR* 677C allele (OR = 0.5; 95% CI = 0.26–0.96) *MTHFD1* 1958A allele (OR =1.9; 95% CI =1.1–3.3)

a *MDS, mothers of Down syndrome individuals; MC, control mothers*.

**Table 2:** **Meta-analyses of studies linking polymorphisms of folate pathway genes to the maternal risk for having a birth with Down syndrome**.

**References**	**MDS/MC^a^**	**Studied polymorphism/s**	**Overall findings (Significant data are in bold)**	**Subgroup analysis (Significant data are in bold)**
Zintzaras, 2007	1.129/1.489	*MTHFR* 677C>T	Allele contrast (OR = 1.18; 95% CI = 0.99–1.40)	Whites**^b^** (OR = 1.04; 95% CI = 0.83–1.30)
	746/888	*MTHFR* 1298A>C	Allele contrast (OR = 1.02; 95% CI = 0.80–1.29)	Whites**^b^** (OR = 1.02; 95% CI = 0.68–1.52)
	559/866	*MTRR* 66A>G	Allele contrast (OR = 1.30; 95% CI = 0.97–1.74)	Whites**^b^** (OR = 1.38; 95% CI = 0.94–2.01)
Medica et al., 2009	1.545/2.052	*MTHFR* 677C>T	**Dominant (OR = 1.40; 95% CI = 1.16–1.70)** **Recessive (OR = 1.35; 95% CI = 1.35–1.78)**	N.A.
	1.007/1.318	*MTHFR* 1298A>C	Dominant (OR = 1.06; 95% CI = 0.85–1.31) Recessive (OR = 1.28; 95% CI = 0.78–2.11)	
	623/936	*MTRR* 66A>G	Dominant (OR = 1.54; 95% CI = 0.98–2.42) **Recessive (OR = 1.57; 95% CI = 1.06–2.31)**	
	439/731	*MTR* 2756A>G	Dominant (OR = 1.04; 95% CI = 0.80–1.35) Recessive (OR = 1.43; 95% CI = 0.65–3.13)	
	354/644	*RFC1* 80G>A	Dominant (OR = 1.32; 95% CI = 0.95–1.82) Recessive (OR = 1.24; 95% CI = 0.67–1.40)	
	367/542	*CBS* 844 ins68	Dominant (OR = 0.97; 95% CI = 0.67–1.40) Recessive (N.A.)	
Amorim and Lima, 2013	1.226/1.533	*MTRR* 66 A>G	**Allele contrast (OR = 1.23; 95% CI = 1.02–1.49)**	N.A.
Costa-Lima et al., 2013	2.101/2.702	*MTHFR* 677C>T	**TT vs. CC (OR = 1.51; 95% CI = 1.22–1.87)**	**Sub-Tropical^c^ (OR = 2.39; 95% CI = 1.62–3.54)** Tropical**^c^** (OR = 1.33; 95% CI = 0.81–2.18) Northern**^c^** (OR = 1.20; 95% CI = 0.89–1.62)
			**CT vs. CC (OR = 1.26; 95% CI = 1.10–1.43)**	**Sub-Tropical^c^ (OR = 1.44; 95% CI = 1.17–1.77)** **Tropical^c^ (OR = 1.33; 95% CI = 1.01–1.76)** Northern**^c^** (OR = 1.06; 95% CI = 0.86–1.30)
Wu et al., 2013	2.806/4.597	*MTHFR* 677C>T	**Allele contrast (OR = 1.22; 95% CI = 1.09-1.38)**	Caucasians**^d^** (OR = 1.11; 95% CI = 0.99–1.25) Asians**^d^** (OR = 1.53; 95% CI = 0.97–2.41) **Others^d^ (OR = 1.24; 95% CI = 1.06–1.44)**
			**Dominant (OR = 1.30; 95% CI = 1.12–1.51)**	Caucasians**^d^** (OR = 1.17; 95% CI = 0.98–1.40) **Asians^d^ (OR = 1.75; 95% CI = 1.08–2.82) Others^d^ (OR = 1.34; 95% CI = 1.10–1.64)**
			**Recessive (OR = 1.26; 95% CI = 1.02–1.56)**	Caucasians**^d^** (OR = 1.12; 95% CI = 0.91–1.37) Asians**^d^** (OR = 1.76; 95% CI = 0.66–4.67) Others**^d^** (OR = 1.23; 95% CI = 0.88–1.74)
	1.854/2.364	*MTHFR* 1298A>C	Allele contrast (OR = 1.03; 95% CI = 0.90–1.17)^h^	Caucasians**^d^** (OR = 0.98; 95% CI = 0.80–1.21) Asians**^d^** (OR = 1.23; 95% CI = 0.60–2.53) Others**^d^** (OR = 1.03; 95% CI = 0.90–1.17)
Coppedè et al., 2013a	1.171/1.402	*MTR* 2756A>G	Allele contrast (OR = 1.08; 95% CI = 0.93–1.25)^h^	Caucasians**^e^** (OR = 1.05; 95% CI = 0.83–1.31) Brazilians**^e^** (OR = 1.20; 95% CI = 0.94–1.54)
Coppedè et al., 2013c	930/1.240	*RFC1* 80G>A	**Allele contrast (OR = 1.14; 95% CI = 1.01–1.30)**	N.A.
			Dominant (OR = 1.09; 95% CI = 0.83–1.43)	N.A.
			**Recessive (OR = 1.27; 95% CI = 1.04–1.57)**	N.A.
Yang et al., 2013	2.458/3.144	*MTHFR* 677C>T	**Allele contrast (OR = 1.28; 95% CI =1.11–1.47)**	**Caucasians^f^ (OR = 1.15; 95% CI = 1.03–1.29)** **Asians^f^ (OR = 1.68; 95% CI = 1.08–2.63)** **Others^f^ (OR = 1.02; 95% CI = 1.02–1.43)**
	1.664/2.027	*MTHFR* 1298A>C	Allele contrast (OR = 1.16; 95% CI = 0.98–1.38)	Caucasians**^f^** (OR = 1.03; 95% CI = 0.83–1.29) Asians**^f^** (OR = 1.41; 95% CI = 0.88–2.27) Others**^f^** (OR = 1.28; 95% CI = 0.94–1.74)
	1.478/2.037	*MTRR* 66A>G	**Allele contrast (OR = 1.22; 95% CI = 1.02–1.46)**	Caucasians**^f^** (OR = 1.24; 95% CI = 0.98–1.58) Asians**^f^** (OR = 1.57; 95% CI = 0.94–2.60) Others**^f^** (OR = 1.01; 95% CI = 0.86–1.19)
	1.038/1.286	*MTR* 2756A>G	Allele contrast (OR = 1.07; 95% CI = 0.91–1.25)	Caucasians**^f^** (OR = 0.99; 95% CI = 0.77–1.29) Asians**^f^** (OR = 0.74; 95% CI = 0.29–1.87) Others**^f^** (OR = 1.13; 95% CI = 0.92–1.38)
	897/1.249	*RFC1* 80G>A	**Allele contrast (OR = 1.16; 95% CI = 1.02–1.31)**	Caucasians**^f^** (OR = 1.21; 95% CI = 0.98–1.58) Asians**^f^** (OR = 0.99; 95% CI = 0.43–2.33) Others**^f^** (OR = 1.14; 95% CI = 0.95–1.38)
	825/1.034	*CBS* 844 ins68	Allele contrast (OR = 1.03; 95% CI = 0.82–1.29)	Caucasians**^f^** (OR = 0.78; 95% CI = 0.46–1.33) Asians**^f^** N.A. Others**^f^** (OR = 1.09; 95% CI = 0.85–1.40)
Coppedè et al., 2014	1.171/1.402	*MTRR* 66A>G	**Allele contrast (OR = 1.26; 95% CI = 1.04–1.51)**	Europeans**^g^** (OR = 1.22; 95% CI = 0.96–1.55) Not Europeans**^g^** (OR = 1.36; 95% CI = 0.94–1.96) Mediterraneans**^g^** (OR = 1.10; 95% CI = 0.94–1.29)
			**Dominant (OR = 1.36; 95% CI = 1.10–1.68)**	**Europeans^g^ (OR = 1.31; 95% CI = 1.01–1.70)** **Not Europeans^g^ (OR = 1.47; 95% CI = 1.02–2.11)** Mediterraneans**^g^** (OR = 1.19; 95% CI = 0.91–1.55)
			**Recessive (OR = 1.32; 95% CI = 1.09–1.62)**	Europeans**^g^** (OR = 1.25; 95% CI = 0.97–1.59) **Not Europeans^g^ (OR = 1.48; 95% CI = 1.06–2.07)** Mediterraneans**^g^** (OR = 1.11; 95% CI = 0.85–1.46)
			The study was restricted to Caucasians	
Victorino et al., 2014	2.223/2.807	*MTHFR* 677C>T	**Allele contrast (OR = 1.25; 95% CI = 1.09–1.44)**	**Caucasians^f^ (OR = 1.17; 95% CI = 1.04–1.31)** Asians^f^ (OR = 1.95; 95% CI = 1.04–1.43) Brazilians^f^ (OR = 1.22; 95% CI = 1.05–1.64)
			**TT vs. CC (OR = 1.46; 95% CI = 1.10–1.94)**	**Caucasians^f^ (OR = 1.28; 95% CI = 1.00–1.65)** Asians**^f^** (OR = 2.49; 95% CI = 0.24–25.8) Brazilians**^f^** (OR = 1.38; 95% CI = 0.95–2.02)
			**CT vs. CC (OR = 1.27; 95% CI = 1.07–1.52)**	Caucasians**^f^** (OR = 1.20; 95% CI = 0.93–1.54) **Asians^f^ (OR = 1.46; 95% CI = 1.05–2.03)** **Brazilians^f^ (OR = 1.30; 95% CI = 1.04–1.62)**
	1.601/1.849	*MTHFR* 1298A>C	Allele contrast (OR = 1.06; 95% CI = 0.91–1.24)^h^	Caucasians**^f^** (OR = 1.04; 95% CI = 0.81–1.33) Asians**^f^** (OR = 1.32; 95% CI = 0.92–1.90) Brazilians**^f^** (OR = 1.02; 95% CI = 0.86–1.22)
Rai et al., 2014	3.098/4.852	*MTHFR* 677C>T	**Allele contrast (OR = 1.22; 95% CI = 1.13–1.31)**^h^	**Asians (OR = 1.53; 95% CI = 1.29–1.82)** **Americans (OR = 1.23; 95% CI = 1.07–1.39)** Europeans (OR = 1.04; 95% CI = 0.93–1.16)
Balduino Victorino et al., 2014	1.311/1.674	*MTR* 2756A>G	Allele contrast (OR = 1.11; 95% CI = 0.97–1.26)^h^	Caucasians**^f^** (OR = 1.04; 95% CI = 0.83–1.31) Brazilians**^f^** (OR = 1.14; 95% CI = 0.96–1.34)
	1.486/2.163	*MTRR* 66 A>G	Allele contrast (OR = 1.18; 95% CI = 0.99–1.40)	Caucasians**^f^** (OR = 1.26; 95% CI = 0.96–1.66) Brazilians**^f^** (OR = 1.00; 95% CI = 0.88–1.14)
			**Dominant (OR = 1.29; 95% CI = 1.09–1.53)**	**Caucasians^f^ (OR = 1.42; 95% CI = 1.08–1.88)** Brazilians**^f^** (OR = 1.14; 95% CI = 0.91–1.42)
			**Recessive (OR = 1.33; 95% CI = 1.03–1.71)**	**Caucasians^f^ (OR = 1.43; 95% CI = 1.13–1.83)** Brazilians**^f^** (OR = 1.02; 95% CI = 0.86–1.22)
	825/1034	*CBS* 844ins68	Allele contrast (OR = 1.07; 95% CI = 0.86–1.34)^h^	Brazilians**^f^** (OR = 1.07; 95% CI = 0.83–1.37)
	497/930	*MTHFD1* 1958G>A	**GA vs. GG (OR = 1.33; 95% CI = 1.01–1.75)**	N.A.
	495/743	*TCN2* 776C>G	Allele contrast (OR =1.27; 95% CI =0.83–1.93)^h^	N.A.

a *MDS, mothers of Down syndrome individuals; MC, control mothers*.

b *Caucasian inhabitants of Europe and North America. Studies in Asian populations, in mixed Brazilian populations, or in inhabitants of the Middle East were scarce for subgrouping*.

c *Tropical Regions: between 23.5°Noth (N) and 23.5° South (S); Sub-Tropical Regions: between 23.5° and 40° N/S; Northern Region: ≥40° N*.

d The authors included in the “Caucasians” subgroup both Europeans, North Americans and inhabitants of the Middle East. Brazilian studies conducted either in mixed Brazilian populations or in individuals of European descent were subgrouped as “Others.”

e *The “Caucasian” subgroup was composed by studies performed in Europeans; The Brazilian subgroup was composed by studies performed in mixed Brazilian populations*.

f The authors included in the “Caucasians” subgroup both Europeans, North Americans and inhabitants of the Middle East. Brazilian studies conducted either in mixed Brazilian populations or in individuals of European descent were subgrouped as “Brazilians.”

g *This study was restricted to Caucasians that were subgrouped as follows: Europeans, all the Europeans; Not Europeans, Americans of European descent; Mediterraneans, European inhabitants of Mediterranean regions*.

h *Similar results observed for dominant, recessive, and/or co-dominant models*.

**Table 3:** **Analysis of markers of one-carbon metabolism and gene-nutrient interactions**.

**References**	**Country**	**MDS/MC^a^**	**Studied markers of one-carbon metabolism**	**Studied polymorphism/s**	**Main findings**
James et al., 1999	USA/Canada	57/50	Plasma hcy Plasma methionine Folate intake^b^ Alcohol intake^b^	*MTHFR* 677C>T	Increased hcy levels in MDS. Increased hcy/methionine ratio in MDS. Increased hcy levels in MDS carriers of the *MTHFR* 677CT or TT genotype. Folate intake from foods was significantly lower than the recommended daily allowance in MDS at conception. Increased reported alcohol intake at conception in MDS.
O'Leary et al., 2002	Ireland	48/192	Plasma hcy Plasma folate Plasma vitamin B12	*MTHFR* 677C>T *MTRR* 66A>G	No difference in hcy levels between MDS and control mothers. Increased hcy levels in control mothers carriers of the *MTHFR* 677TT genotype. Plasma folate and vitamin B12 levels resulted significant predictors of hcy levels.
Chadefaux-Vekemans et al., 2002	France	85/107	Plasma hcy	*MTHFR* 677C>T	No difference in hcy levels between MDS and control mothers. No difference in hcy levels according to *MTHFR* 677C>T genotypes.
Bosco et al., 2003	Italy	63/72	Plasma hcy Plasma folate Plasma vitamin B12	*MTHFR* 677C>T *MTHFR* 1298A>C *MTRR* 66A>G *MTR* 2756A>G	Increased hcy levels in MDS. No difference in folate or vitamin B12 levels between MDS and control mothers. No interaction between hcy, folate or vitamin B12 levels and the studied polymorphisms.
Takamura et al., 2004	Japan	31/60	Plasma hcy Serum folate Serum vitamin B6 Serum vitamin B12	*MTHFR* 677C>T	Increased hcy levels in MDS. Decreased serum folate levels in MDS. No difference in vitamin B6 or B12 levels between MDS and control mothers. No interaction between the *MTHFR* 677C>T polymorphism and markers of one-carbon metabolism.
da Silva et al., 2005	Brazil	154/158	Plasma hcy	*MTHFR* 677C>T *MTHFR* 1298A>C *MTRR* 66A>G *MTR* 2756A>G *CBS* 844 ins68	Increased hcy levels in MDS. Increased hcy levels in MDS carriers of the *MTHFR* 677TT genotype.
Scala et al., 2006	Italy	94/264	Plasma hcy	*MTHFR* 677C>T *MTHFR* 1298A>C *MTRR* 66A>G *MTR* 2756A>G *CBS* 844 ins68 *RFC1* 80G>A *MTHFD1* 1958G>A	No difference in hcy levels between MDS and control mothers. No interaction between hcy levels and the studied polymorphisms.
Martínez-Frías et al., 2006	Spain	91/90	Plasma hcy	*MTHFR* 677C>T *MTHFR* 1298A>C *MTRR* 66A>G	Increased hcy levels in MDS. The *MTHFR* 1298A>C polymorphism was associated with hcy levels. Interactions between *MTHFR* 1298A>C and *MTRR* 66A>G polymorphisms affect hcy levels, but in different ways in MDS than in control mothers.
Wang et al., 2007	China	100/100	Plasma hcy	*MTHFR* 677C>T	Increased hcy levels in MDS. Increased hcy levels in carriers of *MTHFR* 677CT or TT genotypes.
Meguid et al., 2008	Egypt	42/48	Folate intake^b^	*MTHFR* 677C>T *MTHFR* 1298A>C	Folate intake from foods was significantly lower than the recommended daily allowance in MDS.
Biselli et al., 2008b	Brazil	58/49	Plasma hcy	*MTHFR* 677C>T *MTHFR* 1298A>C *MTR* 2756A>G *RFC1* 80A>G	Increased hcy levels in MDS. Interaction between hcy levels and the *MTHFR* 1298A>C polymorphism: hcy levels were higher in MDS carriers of the *MTHFR* 1298CC genotype, but the same genotype resulted in lower hcy levels in control mothers.
Kohli et al., 2008	India	92/91	Plasma hcy Folate intake^b^	*MTHFR* 677C>T	Decreased hcy levels in MDS.
Santos-Rebouças et al., 2008	Brazil	103/108	Folate intake^b^ Methionine^b^ Zinc^b^ Vitamin B6^b^ Vitamin B12^b^	*MTHFR* 677C>T *MTHFR* 1298A>C *MTRR* 66A>G	Folate intake from foods was significantly lower than the recommended daily allowance in both MDS and control mothers. MDS had an estimate lower intake of zinc and methionine respect to control mothers.
Mohanty et al., 2012	India	52/52	Plasma hcy Serum folate Vitamin B12 RBC folate^c^	*MTHFR* 677C>T	Decreased serum folate levels and trend for decreased RBC folate in MDS.
Mendes et al., 2010; Marucci et al., 2012; Zampieri et al., 2012a,b	Brazil (São Paulo)	105/185	Plasma hcy Serum folate Plasma MMA^d^	*MTHFR* 677C>T *MTHFR* 1298A>C *MTHFR* 1317T>C *MTR* 2756A>G *MTRR* 66A>G *RFC1* 80G>A *TCN2* 67A>G *TCN2* 776C>G *CBS* 844ins68 *CBS* 833T>C *MTHFD1* 1958G>A *BHMT* 742G>A *DHFR* 19bp ins/del *SHMT* 1420C>T	*MTHFR* 677CT or TT genotypes were associated with plasma folate levels below the 25^th^ percentile. *RFC1* 80GG genotype was associated with plasma folate levels below the 25^th^ percentile only in women aged ≤ 35 years at conception. *MTRR* 66AG or GG genotypes were associated with MMA concentrations above the 75^th^ percentile.
Wang et al., 2013a	China	104/184	Plasma hcy Serum folate	*MTRR* 524C>T	Increased hcy levels in MDS. The *MTRR* 524TT genotype was associated with low hcy levels in MDS.
Pandey et al., 2013	India	80/100	Plasma hcy Serum folate RBC folate^c^	*MTHFR* 677C>T *MTHFR* 1298A>C	Decreased serum folate and RBC folate, and increased hcy levels in MDS, but no association with the genotype.
Coppedè et al., 2013a, 2014	Italy	172/187	Plasma hcy Plasma folate Plasma vitamin B12	*MTR* 2756A>G *MTRR* 66A>G	No difference in hcy, folate or vitamin B12 levels between groups. The *MTRR* 66GG genotype was associated with increased folate levels. The combined *MTR* 2756AA/*MTRR* 66GG genotype was associated with increased folate levels. The combined *MTR* 2756 GG/*MTRR* 66AA genotype was associated with reduced folate and hcy levels.

a *MDS, mothers of Down syndrome individuals; MC, control mothers*.

b *Intake estimated from food frequency questionnaire*.

c *RBC folate, Red blood cell folate*.

d *MMA, methylmalonic acid, an indicator of the vitamin B12 status*.

## Methods

Major online databases, namely PubMed, Scopus, and Web of Science, were searched up to April 24, 2015 using the following terms: “folate,” “polymorphisms,” “gene variant,” “genetic variant,” “homocysteine,” “congenital heart defects,” and “Down syndrome.” Only peer-reviewed case-control studies showing demographic data of case and control mothers, and tabular data of allele and genotype frequencies, have been considered for this review and included in Tables 1, 3, 4. For articles not written in English, only those published in peer-reviewed journals with an English written abstract clearly showing all the relevant information were included. Meeting abstracts, case-reports, commentaries, academic theses, letters to the editors and review articles with no novel data, were not included. All the available meta-analyses matching the search criteria are included and listed in Table 2, providing that they were written in English language with a clear indication of inclusion and exclusion criteria, as well as available tabular data showing both overall and subgroup analyses.

**Table 4:** **Genetic association studies of folate pathway genes as risk factors for having a child with Down syndrome and congenital heart defects**.

**References**	**Country**	**Cases**	**Studied polymorphism/s**	**Main findings**
Brandalize et al., 2009	Brazil	239 MDS^a^: 90 of DS-CHD^b^ 149 of DS without CHD 16 of DS with GI disease^c^ 223 of DS without GI disease	*MTHFR* 677C>T *MTHFR* 1298A>C	*MTHFR* 677CT or TT increased the risk for CHD: All MDS (OR = 2.07; 95% CI = 1.18–3.61), non users of folic acid supplements: (OR = 2.26; 95% CI = 1.25–4.09), users of folic acid supplements (OR = 1.07; 95% CI = 0.20–5.68). No association of CHD with the *MTHFR* 1298A>C polymorphism. No association of the studied polymorphisms with GI disease risk.
Locke et al., 2010	USA	121 case families: (mother, father, proband with DS-CHD) 122 control families: (mother, father, proband with DS and no CHD)	45 single nucleotide polymorphisms (SNPs) of: *MTHFR, MTR, MTRR, RFC1*, and *CBS* genes	Several *RFC1* SNPs showed association with CHD (AVSD^d^): ORs ranging from 1.3 to 3.8 depending on the model considered. The *MTHFR* 1298A allele was over-transmitted to DS-AVSD individuals and under-transmitted to those with no CHD.
Božović et al., 2011	Croatia	112 DS individuals: (54 DS-CHD and 58 DS without CHD) 221 healthy controls 107 MDS 34 triads: (mother, father, proband)	*MTHFR* 677C>T *MTHFR* 1298A>C	No difference in allele or genotype frequencies between DS-CHD cases and DS cases without CHD, no difference between DS cases and controls, and no association between the presence of either *MTHFR* 677C>T or *MTHFR* 1298A>C polymorphisms in the mother and risk of having a child with DS-CHD.
Elsayed et al., 2014	Egypt	61 mothers of CHD individuals: 25 of DS-CHD 36 of CHD without DS 61 control mothers: no children with DS or CHD	*MTHFR* 677C>T	Increased frequency of the *MTHFR* 677CT genotype in mothers of DS individuals with atrioventricular canal compared to control mothers (OR = 1.21; 95% CI = 1.02–1.43).

a *MDS, mothers of Down syndrome individuals*.

b *DS-CHD, Down syndrome individuals with congenital heart defects*.

c *GI disease, congenital gastrointestinal disease*.

d *AVSD, atrioventricular septal defect*.

## Folate metabolism: an overview

**Figure 1:**
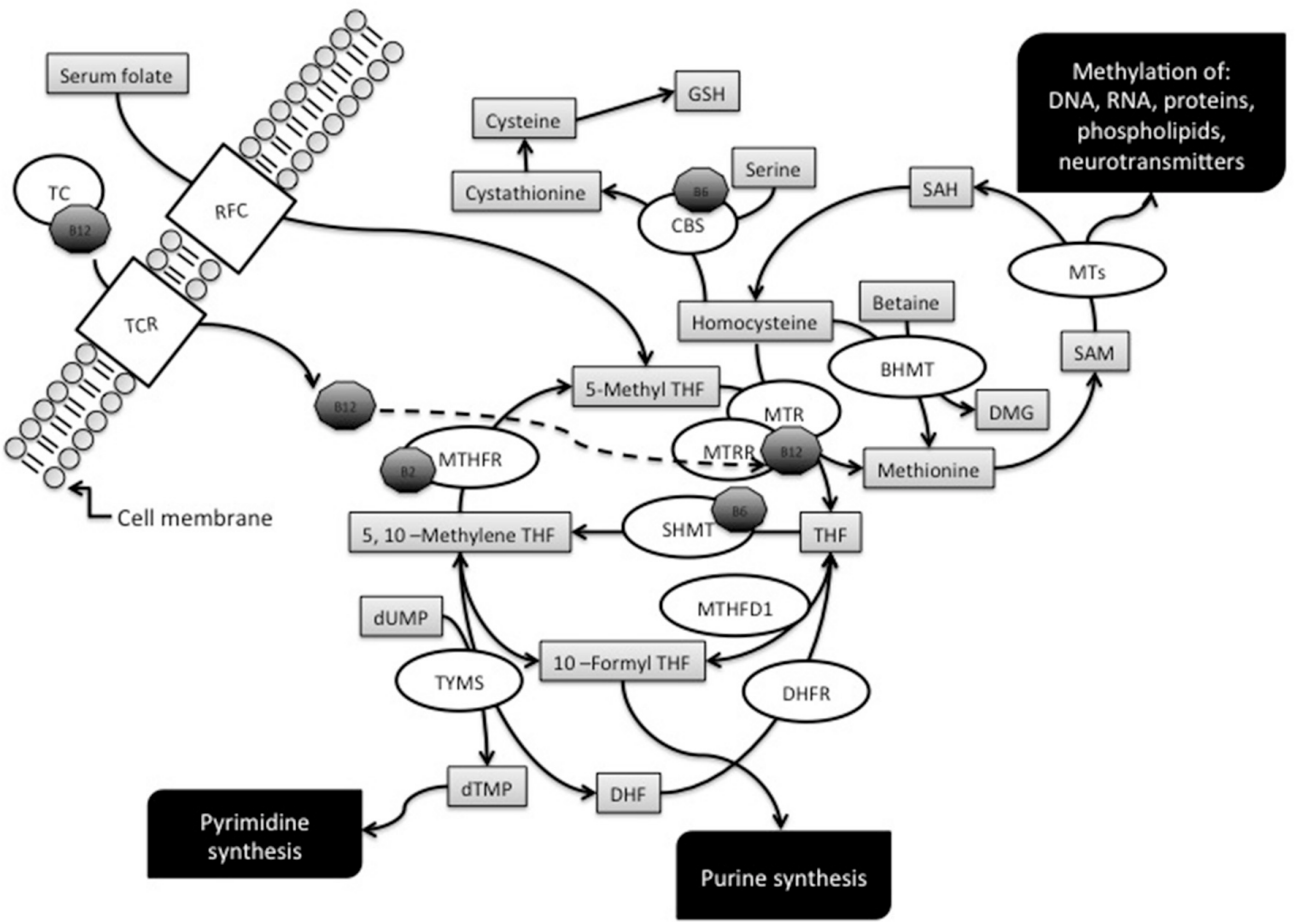
**Overview of the folate metabolic pathway**. The diagram illustrates the enzymes, metabolites and cofactors discussed in this article. Enzymes: BHMT, betaine-homocysteine methyltransferase; CBS, cystathionine β-synthase; DHFR, dihydrofolate reductase; MTs, Methyltransferases; MTHFD1, methylenetetrahydrofolate dehydrogenase; MTHFR, methylenetetrahydrofolate reductase; MTR, methionine synthase; MTRR, methionine synthase reductase; RFC1, reduced folate carrier; SHMT, serine hydroxymethyltransferase; TC, transcobalamin; TCR, tanscobalamin receptor; TYMS, thymidilate synthase. Metabolites: DHF, dihydrofolate; GSH, glutathione; THF, tetrahydrofolate; dTMP, deoxythymidine monophosphate; dUMP, deoxyuridine monophosphate; SAH, *S*-adenosyl homocysteine; SAM, *S*-adenosylmethionine. Cofactors: B2, vitamin B2; B6, vitamin B6; B12, vitamin B12 or cobalamin.

Folate is the general term for a water-soluble B vitamin (vitamin B9) which is naturally found in foods such as green leafy vegetables, liver, beans, egg yolks, cereals, some citric fruits, kiwis, and strawberries (Fenech, 2012). Dietary folates are essential for normal cell growth and replication, since they work as donors and acceptors of one-carbon units during the synthesis of nucleic acids, amino acids, and *S*-adenosylmethionine (SAM), the main intracellular methylating agent (Figure 1). Therefore, a folate restriction results in aberrant cell growth, impaired DNA methylation, and increases the rate of point mutations, chromosome damage, and aneuploidy (Wang et al., 2004; Beetstra et al., 2005; Fenech, 2012). Folic acid is a synthetic compound, structurally similar but with higher bioavailability than naturally occurring folate, which is used in supplements and in fortified foods (Barua et al., 2014). An overview of folate metabolism, also known as one-carbon metabolism, is provided in Figure 1 that illustrates the main enzymes of the pathway whose gene polymorphisms have been investigated as potential maternal risk factors for the birth of a child with DS.

5-Methyltetrahydrofolate (5-methylTHF) is the main form of circulating folate in the plasma and can be transported into the cells by means of folate carriers and receptors, one the best characterized being the ubiquitously expressed reduced folate carrier (RFC), coded by the *SLC19A1* gene (commonly known as *RFC1* gene) (Hou and Matherly, 2014).

Intracellularly, 5-methylTHF functions as a methyl donor for homocysteine (hcy) remethylation, a reaction catalyzed by the methionine synthase (MTR) enzyme that transfers a methyl group from 5-methylTHF to hcy, forming tetrahydrofolate (THF) and methionine. Cobalamin (vitamin B12) is a cofactor in this reaction and methionine synthase reductase (MTRR) is required for the maintenance of MTR in its active state (Guéant et al., 2013). The MTR enzyme is ubiquitously expressed, whereas another hcy remethylation system, mainly expressed in the liver and in the kidneys, involves the betaine-homocysteine methyltransferase (BHMT) enzyme (Obeid, 2013).

Methionine is then converted to SAM by methionine adenosyltransferase, and most of the SAM generated is used in transmethylation reactions whereby it is converted to *S*-adenosylhomocysteine (SAH) by transferring the methyl group to diverse biological acceptors, including proteins and DNA. When the DNA is the final acceptor of the methyl group, the reaction is catalyzed by DNA methyltransferases (DNMTs). SAH is then converted to hcy and adenosine by SAH hydrosylase (Blom and Smulders, 2011). If not converted into methionine, hcy can enter the transsulfuration pathway and be condensed with serine to form cystathionine in a reaction catalyzed by cystathionine β-synthase (CBS), which requires vitamin B6 as a cofactor. Cystathionine is then hydrolyzed to cysteine that, apart for its role in protein synthesis, is the precursor of the antioxidant compound glutathione (GSH) (Blom and Smulders, 2011).

The folate pathway is also pivotal for the synthesis of nucleic acid precursors, and THF, resulting from the reaction catalyzed by MTR, can be directly converted into 5,10-methyleneTHF by the action of serine hydroxymethyltransferase (SHMT). Depending on cellular demands, 5,10-methyleneTHF can be used for thymidylate synthesis, for purine synthesis, or for the production of 5-methylTHF required for hcy remethylation reactions (Figure 1). The tri-functional enzyme methylenetetrahydrofolate dehydrogenase (MTHFD1) interconverts THF derivatives for purine, methionine and thymidylate synthesis. The enzyme consists of three activities: 5,10-methylTHF dehydrogenase, 5,10-methenylTHF cyclohydrolase, and 10-formylTHF synthetase, and catalyzes three sequential reactions in the interconversion of THF derivatives. Once generated by the 10-formylTHF synthetase activity, 10-formylTHF can donate one-carbon groups for purine biosynthesis. Thymidylate synthase (TYMS) converts deoxyuridine monophosphate (dUMP) and 5,10-methyleneTHF to deoxythymine monophosphate (dTMP) and dihydrofolate (DHF) in the *de novo* synthesis of pyrimidines. DHF is then reduced back to THF by dihydrofolate reductase (DHFR) (Coppedè, 2009). However, 5,10-methyleneTHF can also be reduced to 5-methylTHF by methylenetetrahydrofolate reductase (MTHFR), a vitamin B2 (riboflavin) dependent enzyme, which is of great importance for the regulation of available folate derivatives for homocysteine remethylation and DNA methylation reactions (Martínez-Frías, 2008).

The one-carbon metabolic pathway is tightly regulated by intracellular levels of metabolites and cofactors, such as high intracellular SAM levels that activate CBS and inhibit MTHFR, thus shifting hcy levels from transmethylation toward transsulfuration reactions (Finkelstein, 2007). Cobalamin, riboflavin, and vitamin B6 are required as cofactors of several enzymes of the pathway (Figure 1), and their bioavailability is essential for their function, such as for example in the case of vitamin B12 which is a cofactor of MTR in the synthesis of methionine (Guéant et al., 2013). Also cobalamin is obtained from the diet, mainly from meat, eggs, and shellfish, and the absorbed cobalamin forms a complex with transcobalamin (TC, formerly known as transcobalamin II) that transports the vitamin from the bloodstream to the tissues, were cell surface receptors allow it to enter the cells (Fedosov, 2012). The *TCN2* gene codes for TC, and also *TCN2* polymorphisms have been investigated as potential maternal risk factors for the birth of a DS child (Table 1).

## The biology of human female meiosis, the origin of chromosome 21 malsegregation, and the potential contribution of dietary folate availability

In females of the human species, primordial oocytes enter meiosis I (MI) during fetal development, undergo DNA replication and homologous recombination, and then remain arrested in prophase I (dictyotene stage) for several years until ovulation. Meiosis II (MII) is completed only after fertilization that can occur from almost 13 to more than 40 years after the initiation of meiosis I. Advanced maternal age at conception and impaired chromosome 21 recombination represent the two best-known maternal risk factors for chromosome 21 non-disjunction (Lamb et al., 1997; Oliver et al., 2008). Particularly, MI errors are associated with the absence of recombination or with the presence of a single event near the telomere of 21q (Warren et al., 1987; Lamb et al., 1997), and these associations appear to be independent of the age of the oocyte (Oliver et al., 2012). By contrast, MII errors are associated with recombination occurring near the centromere of 21q (Lamb et al., 1997), which appears to depend on the increasing age of the oocyte (Oliver et al., 2012). In this regard it was recently observed that genomic features that affect the accessibility of a specific chromosome region to recombination, including GC content and CpG fraction, are altered in at least a proportion of oocytes with MI and MII errors, and particularly in those with recombinant events occurring in proximal regions, suggesting that factors characteristic of peri-centromeric DNA such as chromatin structure or epigenetic modifications may affect the accessibility of a specific chromosome region to recombination in at least a proportion of oocytes with meiotic errors (Oliver et al., 2014).

Interestingly, a recent screening of more than 20,000 oocytes revealed that most of trisomy 21 cases are due to errors occurred at maternal MI or to sequential MI and MII errors, and should be therefore linked to recombination errors that occurred or initiated during the fetal development of the mother in the maternal grandmother's body (Kuliev et al., 2011). However, more than 35% of the cases were due to maternal MII errors, occurring in adult life. In addition, not all trisomy 21 embryos survive up to the birth, and the aneuploid embryo survival might also depend on the meiotic origin of the error (Kuliev et al., 2011).

**Figure 2:**
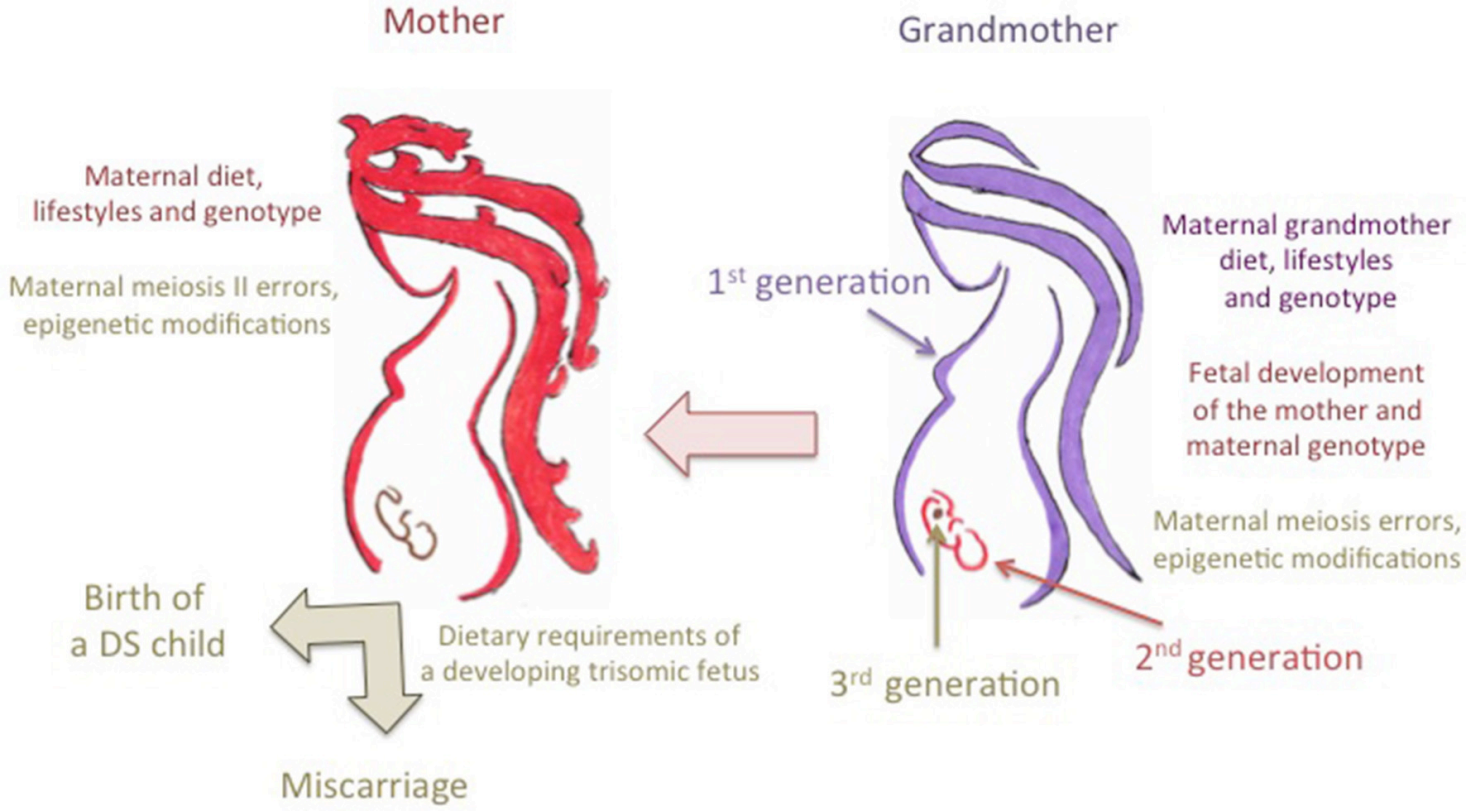
**Trans-generational contribution of folate metabolism to the risk of birth of a child with Down syndrome**. The maternal grandmother's diet during pregnancy provides dietary folates, i.e., the one-carbon units required for the correct development of the mother which is still a developing embryo in the maternal grandmother's body, including those required for the initiation of meiosis of primordial oocytes. Chromosome 21 recombination errors, leading to either meiosis I (MI) or meiosis II (MII) malsegregation events, occur during the prophase of the first meiotic division of primordial maternal oocytes in the maternal grandmother's body. Therefore, the predisposition to those errors is likely to result from complex interactions among the maternal grandmother's dietary provision of folate, her lifestyles such as smoking and drinking that can impair folate metabolism, and both the grandmother's genotype and the genotype of the mother (i.e., the different alleles of folate pathway genes that can account for inter-individual differences in folate absorption and metabolism). The maternal diet and lifestyles at peri-conception and during pregnancy can account for MII errors, as well as for the correct provision of dietary folates to the developing embryo. In this regard, it was hypothesized that complex interactions among maternal diet, lifestyles and genotype, and the metabolic demands of fetuses with trisomy 21, that overexpress several folate pathway genes mapping to chromosome 21, could account for either abortion or survival up to the birth. Those complex gene-environment interactions can also result in epigenetic changes in the developing embryo potentially affecting the birth and/or the complexity of the phenotype.

Despite that inadequate folate intake or impaired metabolism can account for genomic features resulting in aberrant chromosome 21 recombination and malsegregation (Fenech, 2012), we must take into account that, if it is the number and location of the chiasmata between the chromosome 21 homologs that predispose to either MI or MII errors, the predisposition for most chromosome 21 meiotic errors may be set during the prophase of the first meiotic division, during the mother's fetal development in the maternal grandmother's body (Lamb et al., 1997; Coppedè, 2009; Oliver et al., 2012). Therefore, the maternal risk for chromosome 21 malsegregation could be the result of a complex gene-environment interaction involving the maternal grandmother's diet, lifestyles, and genotype and the maternal genotype (Figure 2). Also the maternal diet at peri-conception, that provides dietary folates for the completion of the meiotic process, could be of relevance for chromosome 21 malsegregation (Figure 2). In this regard a recent population-based case-control study compared the use of folic acid-containing supplements among 702 mothers of infants with full trisomy 21 due to maternal nondisjunction and 983 mothers of infants born with no major birth defects. The study revealed that lack of folic acid supplementation during pregnancy might be associated specifically with MII errors in the aging oocyte (OR = 2.00; 95% CI = 1.08–3.71) (Hollis et al., 2013). Indeed, the results of that screening revealed that impaired maternal folate metabolism could be of relevance for the birth of a DS child at advanced maternal age (Hollis et al., 2013).

Collectively, the above-discussed studies suggest that a clear comprehension of the contribution of genetic polymorphisms in folate-pathway genes to the maternal risk of birth of a child with DS should take into account the origin of the meiotic error and, possibly, the dietary habits and lifestyles of at least two generations, i.e., the mother and the maternal grandmother (Figure 2). Unfortunately, those data are largely missing from the studies listed in Table 1, so that at present no meta-analysis of those studies (Table 2) could stratify the data according to MI or MII errors or to maternal age at conception. For what instead concerns the maternal grandmother's diet, only well designed prospective studies will help us to understand if there is a decreased risk for DS in the grandchildren of women taking folic acid supplements.

### Maternal folate metabolism and the survival of a trisomic DS embryo

For what instead concerns the possibility that a trisomic DS embryo will survive up to the birth, a complex maternal-embryonic interaction involving folate metabolism was proposed (Martínez-Frías et al., 2006): Particularly, since many genes involved in folate metabolism map to chromosome 21, including *RFC1*, *CBS*, and others, it was hypothesized that embryos with full trisomy 21 might have a different folate demand than normal embryos, and that maternal folate intake during pregnancy, maternal genotype for genes of the folate metabolic pathway, as well as genotype and expression levels of folate pathway genes mapping to chromosome 21 in the DS embryo, could interact to determine death *in utero* or survival up to the birth (Martínez-Frías et al., 2006) (Figure 2). This is a very interesting hypothesis, but we lack data of comparison between mothers of living DS individuals and women who experience loss of trisomy 21 pregnancies in order to evaluate differences in the frequency of polymorphisms of folate pathway genes between the two groups, or the preferential transmission of certain alleles to those DS embryos that reach the birth with respect to those that die *in utero*, so that the question is still unsolved. However, as discussed in the next sections of this paper, we cannot exclude that maternal polymorphisms of folate pathway genes, their preferential transmission to a DS embryo, as well as epigenetic modifications and expression levels of folate-related genes mapping to chromosome 21, could contribute to the DS phenotype, including the development of congenital defects or that of other diseases later in life.

## Polymorphisms of folate pathway genes and maternal risk of birth of a child with DS

### Methylenetetrahydrofolate reductase (*MTHFR*)

MTHFR catalyzes the reduction of 5,10-methyleneTHF to 5-methylTHF, which is required for the remethylation of hcy to methionine (Figure 1). A common *MTHFR* 677C>T polymorphism (rs1801133), resulting in Ala222Val amino acid substitution, is known to reduce enzyme activity. Indeed, MTHFR works as a dimer protein that is stabilized by physiological levels of folate, and the *MTHFR* 677T allele renders the enzyme thermolabile, particularly in homozygous TT individuals that are prone to dimer destabilization under conditions of reduced folate bioavailability (Guenther et al., 1999; Martínez-Frías, 2008). As a result, the *MTHFR* 677TT genotype has been often associated with hyperhomocysteinemia (Martínez-Frías, 2008). In 1999, James and coworkers reported increased plasma hcy levels and an increased frequency of both *MTHFR* 677CT and TT genotypes in mothers of DS individuals (MDS) with respect to control mothers, and that paper opened the way to the study of folate gene polymorphisms as maternal risk factors for the birth of a child with DS (James et al., 1999). To date, the *MTHFR* 677C>T polymorphism has been investigated in more than 30 small case-control studies as a potential maternal risk factor for having a birth with DS, yielding conflicting results (Table 1). The first meta-analysis, performed in 2007 to overcome the limits of small case-control cohorts, included 1.129 MDS and 1.489 control mothers, reported borderline results, and could neither confirm nor exclude a contribution for this polymorphism (Zintzaras, 2007), but all the subsequent meta-analyses, performed in recent years following the increasing number of available research papers, confirmed association with the maternal risk of birth of a child with DS (Table 2). Particularly, five meta-analyses have been published between 2013 and 2014 to address this issue (Costa-Lima et al., 2013; Wu et al., 2013; Yang et al., 2013; Rai et al., 2014; Victorino et al., 2014), with the most recent including data from 34 case-control studies for a total of 3.098 MDS and 4.852 control mothers (Rai et al., 2014). They mainly differ for the inclusion criteria because, according to the author's mother language, not only papers written in English, but also those written in Chinese or in Spanish and Portuguese languages have been included. However, all those meta-analyses agree that the overall odds ratio (OR) for the birth of a child with DS in carriers of the 677T allele ranges from 1.2 to 1.5 according to the genetic model under investigation, i.e., allele contrast, dominant, recessive, co-dominant, etc. (Table 2). The first meta-analysis in this field also provided the first attempt of data stratification into ethnic groups, and particularly the author performed a subgroup analysis in Caucasians, called “whites” in the original paper, including data from both European and North American studies and observing no significant contribution of the polymorphism in this group (Zintzaras, 2007). Data in populations from Asia, from the Middle East, or in mixed Brazilians were scarce to perform subgroup stratification at that time (Zintzaras, 2007). Subsequent data stratification into ethnic groups revealed that the risk is higher in Asians (OR of about 1.5 or higher, depending on the studied genetic model), and lower in Caucasians and/or other groups (OR usually ranging between 1.0 and 1.4) (Table 2). In those studies inhabitants of North America and of the Middle East were included together with Europeans in the Caucasian subgroup (Wu et al., 2013; Yang et al., 2013; Victorino et al., 2014). For what concerns the studies performed in Brazilian populations, all were performed in mixed Brazilian populations, with the exception of one study (Brandalize et al., 2009) that was performed in Brazilians of European descent, but they were grouped together as “Brazilians” or “others” in the meta-analyses (Wu et al., 2013; Yang et al., 2013; Victorino et al., 2014) (Table 2). Those meta-analyses also revealed that the frequency of the *MTHFR* 677T allele is higher in Caucasian MDS (ranging from 35.6 to 41.5%), followed by Brazilians (ranging from 33.5 to 33.9%), and lower in Asian populations (ranging from 20.0 to 32.3%) (Wu et al., 2013; Victorino et al., 2014). Moreover, when data were stratified according to the geographic origin of the mothers, rather than ethnicity, the higher risk was again observed in Asians (OR = 1.53; 95% CI = 1.29–1.82), followed by Americans (OR = 1.23; 95% CI = 1.07–1.39), and the result was not significant in Europeans (OR = 1.04; 95% CI = 0.93–1.16) (Rai et al., 2014). In that study Brazilians and North Americans were collectively subgrouped as Americans (Rai et al., 2014). Similarly, data stratification according to latitude, revealed a significant effect of the *MTHFR* 677C>T polymorphism in inhabitants of sub-tropical regions (both TT vs. CC and CT vs. CC carriers), followed by tropical regions (only CT vs. CC carriers), but no significant effect was observed for those living in northern regions of the globe (Costa-Lima et al., 2013). Those data reflect complex gene-environment interactions, and are likely to be the result of differences in allele frequencies among different populations coupled with different nutritional habits and exposure to environmental factors, such as sun-light radiations, that could interfere with folate bioavailability (Martínez-Frías, 2008; Coppedè, 2009; Costa-Lima et al., 2013; Rai et al., 2014). We should not forget that the effect of the mutant *MTHFR* 677TT genotype is exacerbated under conditions of low folate intake/bioavailability, but can be counteracted by physiological levels of folate (Martínez-Frías, 2008). Collectively those data reveal the *MTHFR* 677C>T polymorphism represents a weak maternal risk factor for the birth of a child with DS, particularly in those women subjected to nutritional and/or environmental factors resulting in reduced folate bioavailability. Interestingly, we observed association of the *MTHFR* 677C>T polymorphism with both chromosome 13 and 21 malsegregation events in lymphocytes of MDS and matched control mothers, evaluated by means of the micronucleus assay coupled with fluorescence *in situ* hybridization (FISH) to detect aneuploidy (Coppedè et al., 2007, 2009).

Another common *MTHFR* polymorphism, the *MTHFR* 1298A>C one (rs1801131), results in a Glu429Ala aminoacidic change and is in linkage disequilibrium with the *MTHFR* 677C>T one. Particularly, the *MTHFR* 677T-1298C haplotype is rare, and the double homozygous 677TT-1298CC genotype leads to MTHFR protein instability and inactivity, often resulting in prenatal death (Martínez-Frías, 2008). Meta-analysis data revealed that the frequency of the *MTHFR* 1298C allele is higher in Asians (36.0–40.0%), followed by Caucasians (33.0–35.0%) and Brazilians (23.0–25.0%) (Victorino et al., 2014). All the literature meta-analyses performed so far reveal that the *MTHFR* 1298A>C polymorphism is not an independent maternal risk factor for the birth of a child with DS, even after subgroup stratification for ethnicity or geographical factors (Table 2). However, several case-control studies revealed that haplotypes or combined genotypes generated by *MTHFR* 677C>T and 1298A>C polymorphisms increase the maternal risk for a birth of a child with trisomy 21 more than the presence of the *MTHFR* 677C>T one alone, strengthening the evidence of a functional interaction of both polymorphisms on protein stability and activity (Grillo et al., 2002; Acácio et al., 2005; Scala et al., 2006; Brandalize et al., 2009; Zampieri et al., 2012b).

### Methionine synthase and methionine synthase reductase (*MTR* and *MTRR*)

MTR is a cobalamin-dependent enzyme that catalyzes the transmethylation of hcy to methionine and MTRR, a NADPH-dependent diflavin enzyme, is required for the reductive activation of MTR (Figure 1). A common *MTRR* 66A>G polymorphism (rs1801394), resulting in Ile22Met amino acid change, was the second polymorphism of the folate pathway, after the *MTHFR* 677C>T one, to be associated with the maternal risk of having a birth with DS in North American women (Hobbs et al., 2000). Subsequent research papers yielded conflicting results (Table 1), but starting from the meta-analysis conducted in 2009 by Medica and coworkers in 623 MDS and 936 control mothers (Medica et al., 2009), all the following meta-analyses confirmed association of this polymorphism with maternal risk for trisomy 21 in the offspring, with overall ORs ranging from 1.2 to 1.6 depending on the genetic model under investigation (Table 2). Subgroup stratification into ethnic groups, performed by recent meta-analyses (Balduino Victorino et al., 2014; Coppedè et al., 2014), suggests a significant effect in Caucasians under both dominant and recessive genetic models (Table 2). Particularly, for this polymorphism the frequency of the *MTRR* 66G allele ranges from 35.8 to 54.3% among Caucasians, from 40.0 to 48.0% in mixed Brazilian populations, and from 41.5 to 62.5% in Asians (Wang et al., 2008; Balduino Victorino et al., 2014; Coppedè et al., 2014). Furthermore, geographic stratification among Caucasians revealed that the risk is higher in those of European descent that do not live in Europe (OR = 1.47; 95% CI = 1.02–2.11; dominant model) than in European Caucasians (OR = 1.31; 95% CI = 1.01–1.70; dominant model). Furthermore, when the analysis was restricted to the Caucasian inhabitants of Mediterranean regions no significant effect was observed (OR = 1.19; 95% CI = 0.91–1.55; dominant model), suggesting that also for this polymorphism geographic and dietary factors can counteract the negative effect of the genetic background (Coppedè et al., 2014). Recently, another *MTRR* polymorphism, namely *MTRR* 524C>T (rs1532268) resulting in Ser175Leu replacement, has been linked to the maternal risk of birth of a child with DS (Wang et al., 2013a). Particularly, the authors observed decreased maternal risk for carriers of the 524T allele that was associated with reduced hcy levels in Chinese women, but did not search for linkage or interaction with the *MTRR* 66A>G one (Wang et al., 2013a).

Concerning the *MTR* gene, the *MTR* 2756A>G polymorphism (rs 1805087), leading to the Asp919Gly substitution, was the third variant of the folate pathway to be associated with the maternal risk of having a birth with DS (Bosco et al., 2003). However, subsequent case-control studies failed to confirm this association (Table 1), and recent meta-analyses confirmed that the *MTR* 2756A>G polymorphism is not an independent maternal risk factor for a DS offspring (Yang et al., 2013; Coppedè et al., 2013a; Balduino Victorino et al., 2014). The contribution of the *MTR* 2756G allele to the maternal risk of birth of a child with DS was investigated mainly in European and mixed Brazilian populations, with similar reported allele frequencies of about 18–21% (Coppedè et al., 2013a), while the study by Liao et al. (2010) showed allele frequencies of less than 10% in Asians. The functional role of both *MTR* 2756A>G and *MTRR* 66A>G polymorphisms has been largely investigated with regards to their possible contribution to circulating hcy, folate, or vitamin B12 levels, yielding conflicting results (Coppedè, 2009). In this regard, we recently screened a large cohort of MDS and matched control mothers observing that the *MTRR* 66A>G polymorphism, but not the *MTR* 2756A>G one, was associated with increased serum folate levels in GG carriers (Coppedè et al., 2013a, 2014). Others, reported association of *MTRR* AG and GG genotypes with high methylmalonic acid (MMA) concentrations, an indicator of the vitamin B12 status, in Brazilian women (Zampieri et al., 2012b). However, when both polymorphisms were considered simultaneously, carriers of the combined *MTR* 2756AA/*MTRR* 66GG genotype showed increased serum folate levels, while carriers of the *MTR* 2756GG/*MTRR* 66AA genotype had both reduced folate and hcy levels (Coppedè et al., 2014). Several previous papers reported joint effects of polymorphisms of genes of the folate metabolic pathway to circulating levels of B vitamins, hcy or related metabolites, as well as to the overall maternal risk of birth of a child with DS: for example, complex interactions between *MTRR* 66A>G and *MTHFR* 1298A>C polymorphisms significantly affected hcy levels in Spanish MDS and control mothers (Martínez-Frías et al., 2006), and many authors reported association of combined *MTHFR/MTRR* or *MTRR/MTR* genotypes with maternal risk of birth of a DS baby (Hobbs et al., 2000; Bosco et al., 2003; Wang et al., 2008; Coppedè et al., 2009, 2014; Brandalize et al., 2010; Liao et al., 2010) (Table 1).

### Reduced folate carrier (*RFC1*)

The ubiquitously expressed RFC has unequivocally established itself as the major transport system in mammalian cells and tissues for folate cofactors (Hou and Matherly, 2014). A common *RFC1* 80G>A polymorphism (rs1051266), resulting in Arg27His replacement, was suggested to alter folate uptake, and the combined *RFC1* 80GG/*MTHFR* 677TT genotype was associated with increased plasma hcy levels (Chango et al., 2000). In 2006, we observed a borderline significant increased maternal risk of birth of a child with DS for carriers of the combined *RFC1* 80GG/*MTHFR* 677TT genotype, and a reduced risk for carriers of *RFC1* 80(AA or AG)/*MTHFR* 1298AA genotypes (Coppedè et al., 2006). Subsequent studies evaluating the possible contribution of this polymorphism to the maternal risk of having a DS child were conflicting (Table 1), but two recent meta-analyses (Yang et al., 2013; Coppedè et al., 2013c) suggest that it could represent an independent maternal DS risk factor with ORs ranging from 1.1 to 1.3 according to the model under investigation (Table 2). Moreover, despite that its functional role is still controversial, the *RFC1* 80G>A polymorphism has been associated with reduced red cell folate concentrations among healthy women (Stanisławska-Sachadyn et al., 2009), and with reduced serum folate concentrations in MDS (Zampieri et al., 2012b). However, less than 1.000 MDS were available for those meta-analyses, and subgroup stratification yielded inconclusive results likely because of the small case-control cohorts in each ethnic group (Coppedè et al., 2013c; Yang et al., 2013). However, *RFC1* 80G allele frequency resulted higher in Caucasian and Brazilian MDS (ranging between 49.0% and 54.0%) than in Asian ones (36.0–36.5%) (Coppedè et al., 2013a). Therefore, further studies are required to clarify the contribution of this polymorphism to the maternal risk of a DS birth.

### Cystathionine β-synthase (*CBS*)

CBS is a hemoprotein that catalyzes the condensation of hcy and serine to form cystathionine in the transsulfuration pathway (Figure 1). Concerning the *CBS* gene, two common polymorphisms have been investigated as maternal risk factors for the birth of a child with DS (Table 1). The first one consists of the insertion of 68-bp within exon 8 (*CBS* 844ins68, no rs#) that results in the duplication of a splice site at the intron7/exon 8 junction of the gene (Sperandeo et al., 1996), and two recent literature meta-analyses, both performed with 825 MDS and 1.034 control mothers, failed to find association of the *CBS* 844ins68 allele and maternal risk of having a DS child (Yang et al., 2013; Balduino Victorino et al., 2014). The second polymorphism, an 833T>C substitution (rs5742905) leading to an Ile278Thr replacement associated with mild hyperhomocysteinemia (Shao et al., 2005), gave conflicting results as a maternal risk factor for a DS birth in genetic association studies (Zampieri et al., 2012b; Wang et al., 2013b), but has been investigated less extensively than the previous one and no meta-analysis is yet available.

### Methylenetetrahydrofolate dehydrogenase (*MTHFD1*)

MTHFD1 is a trifunctional enzyme of pivotal importance in the interconversion of folate cofactors for either nucleic acid synthesis or hcy remethylation reactions (Figure 1). The *MTHFD1* 1958G>A polymorphism (rs2236225), leading to Arg653Gln, reduces enzyme stability and activity and was first investigated as a maternal risk factor for trisomy 21 in Southern Italian women, showing association with DS risk in combination with the *RFC1* 80G>A polymorphism (the combined *MTHFD1* 1958AA/*RFC1* 80GG genotype) (Scala et al., 2006). Subsequent studies were conflicting (Zampieri et al., 2012a,b; Liao et al., 2014; Izci Ay et al., 2015), but a recent meta-analysis of 497 MDS and 930 control mothers revealed a weak association with maternal risk of DS, but only in GA vs. GG carriers (OR = 1.33; 95% CI = 1.01–1.75) (Balduino Victorino et al., 2014). This is a very interesting data, but results are still preliminary due to the relatively small number of MDS available for the meta-analysis. Indeed, subgroup stratification was not possible (Balduino Victorino et al., 2014), making this variant a candidate allele to be investigated in further studies.

### Transcobalamin (*TCN2*)

In the circulation, TC is the transport protein of cobalamin required for its cellular uptake mediated by specific membrane receptors (TCR) (Figure 1). A common *TCN2* 776C>G polymorphism (rs1801198), results in Arg232Pro replacement and impairs cobalamin metabolism (von Castel-Dunwoody et al., 2005). Genetic association studies revealed associations of this polymorphism with maternal risk of DS birth either alone (Zampieri et al., 2012b), or in combination with *MTR* (combined *TCN2* 776CC/*MTR* 2756AG genotype) (Fintelman-Rodrigues et al., 2009) or *MTHFR* (combined *TCN2* 776CG/*MTHFR* 677TT genotype) (Liao et al., 2014) polymorphisms. However, a recent meta-analysis of 495 MDS and 743 control mothers failed to confirm it as independent maternal risk factor for the birth of a DS baby (Balduino Victorino et al., 2014). Another *TCN2* polymorphism, *TCN2* 67A>G (rs9606756) leading to Ile23Val substitution, was investigated only in Brazilian women and showed no association with maternal risk for a DS birth (Zampieri et al., 2012b).

### Serine-hydroxymethyltransferase (*SHMT*), thymidilate synthase (*TYMS*), and dihydrofolate reductase (*DHFR*)

SHMT uses serine as the one-carbon donor for the conversion of THF into 5,10-methyleneTHF, that can be used for thymidylate synthesis in the reaction catalyzed by TYMS that produces dTMP and DHF, which is then reduced back to THF by DHFR (Figure 1). Polymorphisms of *SHMT, TYMS*, and *DHFR* have been investigated only in one case-control study each (Coppedè et al., 2009; Mendes et al., 2010; Marucci et al., 2012) so that data are still preliminary and no meta-analysis is yet possible. Particularly, the *SHMT* 1420C>T polymorphism (rs1979277), that results in Leu474Phe replacement and impairs SHMT nuclear transport and subsequent thymidilate synthesis (Woeller et al., 2007), was investigated in 105 MDS and 185 control mothers from Brazil, and both 1420CC (OR = 0.35; 95% CI = 0.2–0.6) and 1420CT (OR = 0.2; 95% CI = 0.1–0.4) genotypes resulted in a decreased maternal risk of birth of a child with DS with respect to the 1420TT genotype (Marucci et al., 2012). We investigated two common *TYMS* polymorphisms in 94 MDS and 113 control mothers of Italian origin (Coppedè et al., 2009), namely a 28-bp short tandem repeats (rs34743033) in the 5′-untranslated region (5′-UTR) that is linked to gene expression levels (Horie et al., 1995), and a 6-bp deletion (1494 ins/del) polymorphism in the 3′-UTR (rs34489327) that affects mRNA stability into the cytoplasm (Ulrich et al., 2002). None of the two studied *TYMS* polymorphisms resulted an independent maternal risk factor for a DS birth, but the combined *MTHFR* 1298AC/*TYMS* 28-bp 2R/2R genotype resulted in decreased maternal risk (Coppedè et al., 2009). Concerning the *DHFR* gene, Mendes and coworkers (2010) genotyped 105 MDS and 185 control mothers from Brazil for the presence of a 19-bp ins/del polymorphism (rs70991108) that was previously linked to alterations in folate metabolism (Kalmbach et al., 2008), but no association with maternal risk of a DS birth was observed (Mendes et al., 2010). Unfortunately, due to the absence of replication studies, none of these polymorphisms can be confirmed or excluded as a maternal risk factor for having a birth with trisomy 21.

### Betaine-homocysteine methyltransferase (*BHMT*)

BHMT is a zinc-dependent protein that catalyzes the synthesis of methionine from the methyl-donor betaine and hcy (Figure 1), thus contributing to the regulation of homocysteine levels, and is particularly active in the liver and in the kidneys (Obeid, 2013). A *BHMT* 742G>A polymorphism (rs3733890) leads to Arg239Gln substitution and to a slight reduction in enzyme kinetics, that however do not appear to alter circulating hcy levels (Li et al., 2008). However, three recent case-control studies performed in Brazilian women (Zampieri et al., 2012a,b; Amorim et al., 2013) suggest that carriers of the *BMTH* 742A allele, and particularly homozygous 742AA carriers, are at decreased maternal risk of birth of a child with DS than 742GG carriers (Table 1), making this gene a valuable candidate for further investigation.

### DNA methyltransferase 3B (*DNMT3B*)

In their pioneering studies James et al. (1999) and Hobbs et al. (2000) advanced the hypothesis that polymorphisms of folate pathway genes could result in altered methylation of chromosome 21 peri-centromeric regions, favoring its malsegregation during maternal meiosis. Particularly, to support a causal association between DNA hypomethylation, peri-centromeric decondensation, and abnormal chromosome segregation, Hobbs et al. (2000) reported the example of the monogenic disease, Immunodeficiency, Centromeric instability and Facial anomalies (ICF) syndrome, which is a Mendelian disorder associated with *DNMT3B* mutations and DNA methylation defects of satellite and non-satellite regions (Hobbs et al., 2000). Despite that a direct evidence of a link between folate pathway gene polymorphisms and DNA methylation levels of chromosome 21 peri-centromeric regions has not yet been demonstrated, two recent genetic association studies suggest that promoter polymorphisms of the *DNMT3B* gene might be associated with the maternal risk of birth of a child with DS (Coppedè et al., 2013b; Jaiswal et al., 2015). DNMT3b is a *de novo* DNMT that localizes primarily to the nucleus, and is required for the regulation of DNA methylation at centromeric and peri-centromeric regions of human chromosomes (Gopalakrishnan et al., 2009). *DNMT3B* promoter polymorphisms, namely -149C>T (rs2424913) and -579G>T (rs1569686), are in strong linkage disequilibrium, give rise to functional haplotypes that could impair protein expression levels, and have been associated to various kinds of human cancers (Zhu et al., 2012). We genotyped 172 MDS and 157 control mothers of Italian origin observing a decreased risk of birth of a child with DS in carriers of the *DNMT3B* -579T allele, with respect to GG carriers; moreover, the combined *DNMT3B* -579GT/-149CC genotype was associated with an even more significant reduced maternal risk of birth of a child with DS (Table 1) (Coppedè et al., 2013b). More recently, a similar study performed in 150 Indian MDS and 172 control mothers revealed a significant increased frequency of the *DNMT3B* -579G/-149T haplotype in the MDS group (Jaiswal et al., 2015). Overall, both studies suggest that combinations of *DNMT3B* promoter polymorphisms might be associated to the maternal risk for having a birth with trisomy 21, however data are still preliminary and should be confirmed in future studies.

### Gene-gene interactions

Table 1 clearly shows that many authors reported an increased maternal risk of birth of a child with DS for carriers of combined genotypes given by the presence of two simultaneous risk alleles, mainly combinations of *MTHFR* 677T and 1298C alleles, or combinations of *MTHFR* 677T and *MTRR* 66G ones (Table 1). However, when authors searched for the simultaneous presence of more than two risk alleles, they often reported increased maternal risk for a DS birth with the increasing number of variants in folate pathway genes carried by the mother: for example, da Silva et al. (2005) observed that MDS tend to present more variant alleles than control mothers among *MTHFR* 677C>T, *MTHFR* 1298A>C, *MTR* 2756A>G, *MTRR* 66A>G, and *CBS* 844ins68 polymorphisms, and the presence of each uncommon allele was associated with an increment of 25.9% in the risk of a DS pregnancy (da Silva et al., 2005). Similarly, Biselli et al. (2008b) observed that the presence of three or more variant alleles among *MTHFR* 677C>T, *MTHFR* 1298A>C, *MTR* 2756A>G, and *RFC1* 80G>A, resulted in a 1.7-fold increase in maternal risk for a DS birth (Biselli et al., 2008b), while Brandalize et al. (2010) analyzed the genotypes generated by the following polymorphisms: *MTR* 2756A>G, *MTRR* 66A>G, *CBS* 844ins68, and *RFC1* 80G>A, observing that the OR for having a child with DS ranged from 4.8 to 6.9 depending on the number of risk genotypes considered (Brandalize et al., 2010). We used artificial neural networks (ANNs) to discriminate between MDS and control mothers using genotyping data and the frequency of chromosome damage and malsegregation in peripheral lymphocytes evaluated by means of the micronucleus assay (Coppedè et al., 2010). ANNs revealed that micronucleus frequency and genotyping data for *MTHFR* 677C>T, *TYMS* 28bp repeats, *TYMS* 1494 6bp ins/del, *MTR* 2756A>G, and *RFC1* 80G>A polymorphisms, allowed to discriminate between MDS and control mothers with 90% accuracy (Coppedè et al., 2010). Overall, those data suggest that the combined presence of several variant alleles of folate pathway genes is likely to increase significantly the maternal risk of birth of a child with DS.

### Gene-nutrient interactions

In their pioneering study James et al. (1999) not only observed an increased frequency of *MTHFR* 677CT and TT genotypes, but also increased hcy levels, and association of those genotypes with increased hcy levels in MDS with respect to control mothers. Furthermore, the authors noticed an impaired hcy/methionine ratio in MDS, and estimated from food questionnaires that MDS had lower folate intake than the recommended daily allowance at the time of conception (James et al., 1999). In addition, alcohol intake estimated from the questionnaires, which is known to impair MTR activity and hcy to methionine conversion, was higher in MDS than in control mothers (James et al., 1999). Overall, the authors suggested complex gene-environment interactions at the basis of the maternal susceptibility for the birth of a child with DS, and many others have subsequently measured or estimated biomarkers of one-carbon metabolism in MDS and control mothers, including plasma hcy, plasma or serum folate, vitamin B6 and/or vitamin B12, red blood cell (RBC) folate, and related micronutrients, searching for association with the genetic polymorphisms of folate pathway genes (Table 3). Despite that those studies are often conflicting, most of them reported increased hcy levels or decreased measured or estimated folates in MDS with respect to control mothers (Table 3), and several authors observed association with the studied polymorphisms, including *MTHFR* 677C>T and 1298A>C polymorphisms that were often associated with increased hcy levels (James et al., 1999; O'Leary et al., 2002; da Silva et al., 2005; Martínez-Frías et al., 2006; Wang et al., 2007; Biselli et al., 2008b; Zampieri et al., 2012b). Similarly, the *MTRR* 66A>G polymorphism was linked to MMA levels, an indicator of the vitamin B12 status (Zampieri et al., 2012b), with hcy levels in interaction with the *MTHFR* 1298A>C polymorphism (Martínez-Frías et al., 2006), and with both folate and hcy levels in combination with the *MTR* 2756A>G one (Coppedè et al., 2014). The *MTRR* 524C>T polymorphism was associated with reduced hcy levels in carriers of the *MTRR* 524TT genotype (Wang et al., 2013a), and the *RFC1* 80GG genotype was associated with high plasma folate levels in women aged 35 years or less at conception (Zampieri et al., 2012b). In summary, all the three genetic polymorphisms (*MTHFR* 677C>T, *MTRR* 66A>G, and *RFC1* 80G>A) which have been linked to the maternal risk for the birth of a child with DS by literature meta-analyses (Table 2), and other polymorphisms in the same genes (*MTHFR* 1298A>C and *MTRR* 524C>T), have been associated with hematochemical markers of one-carbon metabolism (Table 3). Limitations of those studies, that could also partially account for the conflicting nature of the findings, include the fact that in most of the cases the hematochemical markers have been measured after the birth of DS or healthy children, and do not represent the nutritional status at the time of peri-conception (reviewed in Coppedè, 2009). In this regard, some authors made attempts to estimate the nutritional status at peri-conception by means of food questionnaires (James et al., 1999; Kohli et al., 2008; Meguid et al., 2008; Santos-Rebouças et al., 2008) and others evaluated RBC folate levels that are more useful to monitor the long-term folate status, with respect to serum or plasma folate levels that are only indicators of recent folate intake (Mohanty et al., 2012; Pandey et al., 2013). Data on alcohol consumption at peri-conception are however scarce from those studies (James et al., 1999), and this is another factor that could interfere with one-carbon metabolism, as well as smoking habits or information on other micronutrients, such as for example zinc which may affect the activities of enzymes such as BHMT and MTR (Santos-Rebouças et al., 2008; Fenech, 2012). Taken overall, the studies listed in Table 3 suggest that certain polymorphisms in folate pathway genes are associated with the levels of hematochemical markers of one-carbon metabolism, and that certain MDS could have been under conditions of folate restriction and/or increased hcy levels at the time of conception. However, the complex link between the risk of birth of a child with DS and the intake of dietary folate and related vitamins and micronutrients at peri-conception could be clarified only by future and well-designed prospective studies on large cohorts of individuals. In this regard, it will be also of fundamental importance to take into account that the nutritional status and requirements of three different generations (Figure 2), namely the maternal grandmother, the mother, and the developing DS embryo, coupled with the presence of polymorphisms of folate pathway genes, with the exposure to factors such as alcohol drinking and cigarette smoking during pregnancy, and with the nature of the meiotic error leading to chromosome 21 malsegregation, could interact to determine not only the formation of an embryo with trisomy 21, but also which DS embryo will survive up to the birth (Martínez-Frías et al., 2006; Coppedè, 2009).

Unfortunately, none of the available meta-analyses listed in Table 2 made subgroup comparison between countries with and without mandatory folic acid fortification of foods. Since 1998, fortification of grain products with folic acid has been mandated in the United States of America and subsequent studies revealed that this resulted in changes in the birth prevalence of neural tube defects (NTDs), with modest or no effect in reducing the birth prevalence of DS individuals (Simmons et al., 2004; Canfield et al., 2005), and similar results were observed in other countries after fortifying wheat flour with folic acid (Castilla et al., 2003; Goh et al., 2006). Unfortunately, the two available North American studies addressing the contribution of *MTHFR* 677C>T and/or *MTRR* 66A>G polymorphisms to the maternal risk for having a child with DS were published in 1999 and 2000 (James et al., 1999; Hobbs et al., 2000) and therefore, despite that both observed significant associations between the studied polymorphisms and maternal risk for DS, women enrolled in those studies were mainly collected from 1989 to 1998 and are unlikely to have benefited from the fortification (Hobbs et al., 2000). However, as discussed in Section “The Biology of Human Female Meiosis, the Origin of Chromosome 21 Malsegregation, and the Potential Contribution of Dietary Folate Availability,” a recent case-control study conducted by Hollis and coworkers at six recruitment sites in the United States from 2000 through 2004 (Hollis et al., 2013) revealed that lack of folic acid fortification at peri-conception resulted in increased risk for DS cases due to maternal MII errors in women older than 35 years. Unfortunately, the contribution of folate pathway gene polymorphisms was not evaluated in that study (Hollis et al., 2013). Brazil is another country with mandatory folic acid fortification since 2004, but recent meta-analyses revealed a significant contribution of the *MTHFR* 677T allele as maternal risk factor for the birth of a child with DS in Brazilian women (Yang et al., 2013; Victorino et al., 2014). By contrast, the fortification is not mandatory in Europe and in most Asian countries (Choi et al., 2014). However, taking into account the possible contribution of the maternal grandmother's diet to the maternal risk of birth of a child with DS (Figure 2), it is likely that a better comprehension of the effect of the mandatory fortification adopted by several countries will be possible only in the next generations.

## Maternal polymorphisms of folate pathway genes and risk of congenital heart defects in the DS child

Several congenital complications are observed in individuals with DS (for a review see Weijerman and de Winter, 2010), some of which potentially affected by impaired maternal one-carbon metabolism and consequent epigenetic changes during embryogenesis or impaired requirements of DNA precursors for cellular divisions. However, in this review article the author will focus on congenital heart defects, due to an increasing evidence of a possible contribution of polymorphisms in folate-pathway genes to their occurrence in the DS offspring (Table 4) coupled with scarce or absence of data for other DS comorbidities. The prevalence of CHD in neonates with DS ranges from 43 to 58% worldwide, with atrioventricular septal defect (AVSD) and ventricular septal defect (VSD) being the most common forms of all CHD in children with DS (Weijerman and de Winter, 2010). Quite recently, a population-based case-control study, which included 1.011 MDS that reported their use of supplements containing folic acid at peri-conception, showed that lack of maternal folic acid supplementation was more frequent among infants with DS and AVSD (OR = 1.69; 95% CI = 1.08–2.63) or atrial septal defects (OR = 1.69; 95% CI = 1.11–2.58) than among infants with DS and no heart defect, suggesting that lack of maternal folic acid supplementation is associated with septal defects in infants with DS (Bean et al., 2011). Unfortunately, only a few case-control studies have been conducted so far to evaluate polymorphisms in folate-pathway genes as risk factors for CHD occurrence in a DS birth (listed in Table 4), and no meta-analysis of the data is yet possible. Brandalize et al. (2009) genotyped 239 Brazilian MDS for *MTHFR* 677C>T and 1298A>C polymorphisms, including 90 mothers of a DS child with CHD (DS-CHD) and 149 mothers of a DS child without CHD. The authors observed that MDS carriers of the *MTHFR* 677CT or TT genotype had an increased risk for DS-CHD in the offspring (OR = 2.07; 95% CI = 1.18–3.61). However, when MDS were stratified according to folic acid supplementation at peri-conception, the risk for DS-CHD remained significant only in those with *MTHFR* 677CT or TT genotype that did not take folic acid supplements (OR = 2.26; 95% CI = 1.25–4.09), but was lost in those MDS that used folic acid supplements at peri-conception (OR = 1.07; 95% CI = 0.20–5.68). No association with DS-CHD risk was observed for the *MTHFR* 1298A>C polymorphism (Brandalize et al., 2009). Locke et al. performed a case-control study in a group of 121 American case families (mother, father, and proband with DS and AVSD) and 122 American control families (mother, father, and proband with DS and no CHD), all genotyped for 45 polymorphisms in *MTHFR, MTR, MTRR, RFC1*, and *CBS* genes, observing that several *RFC1* polymorphisms, all in strong linkage with the *RFC1* 80G>A one, showed nominally significant associations with AVSD, with ORs of between 1.34 and 3.78. In addition, the *MTHFR* 1298A allele was over-transmitted to DS individuals with AVSD and under-transmitted to those with no CHD (Locke et al., 2010). By contrast, Božović et al. (2011) genotyped 112 DS subjects (54 with CHD and 58 without CHD) and 221 matched controls from Croatia for both *MTHFR* 677C>T and 1298A>C polymorphisms, observing no difference in allele or genotype frequencies for both polymorphisms between DS-CHD cases and DS cases without CHD, or between DS cases and healthy controls (Božović et al., 2011). Furthermore, the authors genotyped 107 MDS and 34 complete parent–offspring triads. No association was observed between the presence of either *MTHFR* 677C>T or *MTHFR* 1298A>C polymorphism in the mother and risk of DS-CHD offspring, and all MDS did not use folic acid supplements at peri-conception; similarly, the allele transmission of the two *MTHFR* polymorphisms showed no deviations from random segregation, leading the authors to conclude that none of the studied polymorphisms was associated with CHD risk in their cohort (Božović et al., 2011). More recently, Elsayed et al. (2014) genotyped 61 Egyptian mothers of children with septal defects (25 with DS and 36 without DS) and 61 control mothers (with no children with DS or CHD) who did not receive folic acid supplementation in the peri-conceptional period. All women were genotyped for the *MTHFR* 677C>T polymorphism, and the *MTHFR* 677CT genotype resulted significantly higher in mothers of DS with atrioventricular (AV) canal compared to control mothers (OR: 1.21, 95%CI: 1.02–1.43). Collectively those papers are conflicting but four out of five of them (Brandalize et al., 2009; Locke et al., 2010; Bean et al., 2011; Elsayed et al., 2014) suggest a possible contribution of folate metabolism to the development of CHD in DS individuals.

Despite that a meta-analysis of the few studies of DS-CHD is yet not possible due to their relatively small number (Table 4), a recent meta-analysis of 18 case-control studies, that included data from 18.500 cases of CHD, revealed that maternal folic acid supplementation is associated with a significant decreased risk of CHD (OR: 0.72, 95%CI: 0.63–0.82), and the association remained significant even after stratification of the data into CHD subtypes (Feng et al., 2015). Similarly, recent literature meta-analyses of non DS-CHD cases revealed association of maternal *MTHFR* 677C>T and *MTRR* 66A>G polymorphisms with CHD risk in the offspring (Wang et al., 2013c; Cai et al., 2014), leading authors to hypothesize that methylation profiles and other epigenetic abnormalities could contribute to the etiology of heart malformations.

In this regard, Obermann-Borst et al. (2011) performed a study in 143 Dutch children with CHD and 186 healthy children, observing that in the overall CHD group, blood SAM levels, serum folate, and RBC folate levels were significantly higher than in the controls. However, when the analysis was restricted to the subgroup of children with DS-CHD, they showed significantly lower SAM:SAH ratio (the DNA methylation potential) than other CHD cases, suggesting that cases of DS-CHD may be associated with global hypomethylation (Obermann-Borst et al., 2011). A recent study was performed to explore the global methylation profile of fetal heart DNA from 22 medically terminated pregnancies (4 with normal development, 6 with DS-CHD, 6 with DS without CHD, and 6 with isolated CHD) in comparison to blood DNA from 656 subjects (Serra-Juhé et al., 2015). The study revealed remarkably different profiles between tissues, with 407 genes hypomethylated in heart tissue compared to blood, and 339 genes hypomethylated in blood compared to heart tissue. Among the 22 heart DNA samples significant differences in DNA methylation profiles were seen between fetuses with DS and fetuses with a normal karyotype. The comparison of individual DS-CHD cases with two control groups (normal development and DS without CHD) revealed 19 differentially methylated regions. Several epimutations were detected in candidate genes involved in growth regulation, apoptosis and folate pathway in cases of syndromic and isolated CHD, providing evidence that impaired DNA methylation occurred in their developing heart tissue, likely contributing to the onset of CHD (Serra-Juhé et al., 2015).

## Discussion and conclusions

Despite that DS occurs in almost 1 on 700 live births, the molecular mechanisms leading to maternal chromosome 21 malsegregation during oogenesis are still not fully understood. Since 1999, it was suggested that maternal polymorphisms in folate pathway genes could contribute to the epigenetic regulation of the chromatin structure in those regions, thus acting as maternal risk factors for the birth of a child with DS (James et al., 1999). That hypothesis stimulated considerable research over the last 15 years, leading to the production of more than 50 case-control studies that, despite the conflicting nature of their results, revealed a complexity of gene-gene and gene-nutrient interactions in folate metabolism as potential maternal risk factors for having a birth with trisomy for chromosome 21 (Table 1). More recent meta-analyses of those studies have revealed that at least three of the most frequently studied polymorphisms, namely *MTHFR* 677C>T, *MTRR* 66A>G, and *RFC1* 80G>A, could act as weak independent maternal risk factors for having a child with DS, with slight differences among populations likely due to the existence of complex gene-gene and gene-environment interactions (Table 2). Limitations of those studies lies mainly in the fact that, with the exception of the most frequently studied *MTHFR* 677C>T polymorphism for which data on over 3.000 MDS and almost 5.000 control mothers were available for meta-analysis (Rai et al., 2014), data on all the other polymorphisms are available for less than 2.000 MDS, and often less than 1.000 MDS, limiting the power to detect association also during meta-analysis (Table 2). Furthermore, data concerning the occurrence of the meiotic error (MI or MII), the maternal age at conception, as well as smoking and drinking habits, are often unavailable in case-control studies, making it impossible to evaluate their contribution during the meta-analysis (Table 2). However, taking collectively the literature produced so far in this field, we can argue that there is substantial evidence indicating that maternal impairments in folate metabolism could favor at least certain errors leading to chromosome 21 malsegragation; but considering that maternal meiosis in a female starts during embryogenesis, the contribution of the maternal grandmother's folate intake is still a matter of debate (Coppedè, 2009).

Furthermore, overexpression of folate pathway genes mapping to chromosome 21 in a developing DS embryo could result in a different requirement of dietary folates with respect to a normal embryo and, if not properly provided by the maternal diet, could contribute to death *in utero*, impaired growth and, if the birth is reached, to epigenetic changes of several genes likely contributing to the onset of either congenital defects or other diseases in adult-life (Martínez-Frías et al., 2006). Unfortunately, our understanding of this is still in its infancy, due to scarce availability of comparison data between women who experience loss of trisomy 21 pregnancies and those with trisomic fetuses that survive up to the birth, as well as to a few epigenetic studies in DS individuals. In this regard, evidence is accumulating concerning the possible contribution of impaired maternal folate metabolism and epigenetic changes during embryogenesis to the occurrence of DS-CHD cases (Table 4). We cannot exclude that similar mechanisms could also account for other DS comorbidities, but data are still scarce or missing. For example, Brandalize et al. (2009) investigated whether or not maternal *MTHFR* polymorphisms could account also for the occurrence of congenital gastrointestinal disease in DS births observing no association (Table 4), but they only had 16 DS cases with gastrointestinal disease in their cohort (Brandalize et al., 2009). Similarly, recent studies in DS individuals suggest that epigenetic deregulation of several genes could not only account for CHD (Serra-Juhé et al., 2015), but also for other complications such as learning and memory deficits (Dekker et al., 2014) or accelerated aging (Horvath et al., 2015), but how much of this results from *in utero* exposure is still unclear.

In conclusion, the available literature in this field suggests that it is now time for the design of large-scale prospective studies, spanning over at least three different generations, in order to clarify the possible contribution of both maternal and maternal grandmother's diet and genotype to the occurrence of meiotic errors favoring chromosome 21 malsegregation and the birth of a DS child, as well as to other metabolic changes and/or epigenetic modifications leading to death *in utero* or to the occurrence of either congenital or age-related DS comorbidities.

### Conflict of interest statement

The author declares that the research was conducted in the absence of any commercial or financial relationships that could be construed as a potential conflict of interest.
